# *Drosophila melanogaster* Hox Transcription Factors Access the RNA Polymerase II Machinery through Direct Homeodomain Binding to a Conserved Motif of Mediator Subunit Med19

**DOI:** 10.1371/journal.pgen.1004303

**Published:** 2014-05-01

**Authors:** Muriel Boube, Bruno Hudry, Clément Immarigeon, Yannick Carrier, Sandra Bernat-Fabre, Samir Merabet, Yacine Graba, Henri-Marc Bourbon, David L. Cribbs

**Affiliations:** 1 Centre de Biologie du Développement, CBD, UMR5547 CNRS/UPS, Université de Toulouse, Toulouse, France; 2 Institut de Biologie du Développement de Marseille Luminy, IBDML, UMR6216 CNRS, Université de la méditerranée, Marseille, France; The University of North Carolina at Chapel Hill, United States of America

## Abstract

Hox genes in species across the metazoa encode transcription factors (TFs) containing highly-conserved homeodomains that bind target DNA sequences to regulate batteries of developmental target genes. DNA-bound Hox proteins, together with other TF partners, induce an appropriate transcriptional response by RNA Polymerase II (PolII) and its associated general transcription factors. How the evolutionarily conserved Hox TFs interface with this general machinery to generate finely regulated transcriptional responses remains obscure. One major component of the PolII machinery, the Mediator (MED) transcription complex, is composed of roughly 30 protein subunits organized in modules that bridge the PolII enzyme to DNA-bound TFs. Here, we investigate the physical and functional interplay between *Drosophila melanogaster* Hox developmental TFs and MED complex proteins. We find that the Med19 subunit directly binds Hox homeodomains, *in vitro* and *in vivo*. Loss-of-function *Med19* mutations act as dose-sensitive genetic modifiers that synergistically modulate Hox-directed developmental outcomes. Using clonal analysis, we identify a role for Med19 in Hox-dependent target gene activation. We identify a conserved, animal-specific motif that is required for Med19 homeodomain binding, and for activation of a specific Ultrabithorax target. These results provide the first direct molecular link between Hox homeodomain proteins and the general PolII machinery. They support a role for Med19 as a PolII holoenzyme-embedded “co-factor” that acts together with Hox proteins through their homeodomains in regulated developmental transcription.

## Author Summary

Mutations of Hox developmental genes in the fruit fly *Drosophila melanogaster* may provoke spectacular changes in form: transformations of one body part into another, or loss of organs. This attribute identifies them as important developmental genes. Insect and vertebrate Hox proteins contain highly related homeodomain motifs used to bind to regulatory DNA and influence expression of developmental target genes. This occurs at the level of transcription of target gene DNA to messenger RNA by RNA polymerase II and its associated protein machinery (>50 proteins). How Hox homeodomain proteins induce fine-tuned transcription remains an open question. We provide an initial response, finding that Hox proteins also use their homeodomains to bind one machinery protein, Mediator complex subunit 19 (Med19) through a Med19 sequence that is highly conserved in animal phyla. Med19 mutants isolated in this work (the first animal mutants) show that Med19 assists Hox protein functions. Further, they indicate that homeodomain binding to the Med19 motif is required for normal expression of a Hox target gene. Our work provides new clues for understanding how the specific transcriptional inputs of the highly conserved Hox class of transcription factors are integrated at the level of the whole transcription machinery.

## Introduction

The finely regulated gene transcription permitting development of pluricellular organisms involves the action of transcription factors (TFs) that bind DNA targets and convey this information to RNA polymerase II (PolII). Hox TFs, discovered through iconic mutations of the *Drosophila melanogaster* Bithorax and Antennapedia Complexes, play a central role in the development of a wide spectrum of animal species [1], [2]. Hox proteins orchestrate the differentiation of morphologically distinct segments by regulating PolII-dependent transcription of complex batteries of downstream target genes whose composition and nature are now emerging [3]–[7]. The conserved 60 amino acid (a.a.) homeodomain (HD), a motif used for direct binding to DNA target sequences, is central to this activity. Animal orthologs of the *Drosophila* proteins make use of their homeodomains to play widespread and crucial roles in differentiation programs yielding the very different forms of sea urchins, worms, flies or humans [8]. They do so by binding simple TAAT-based sequences within regulatory DNA of developmental target genes [9]–[14]. One crucial aspect of understanding how Hox proteins transform their versatile but low-specificity DNA binding into an exquisite functional specificity involves the identification of functional partners. Known examples include the TALE HD proteins encoded by *extradenticle* (*exd*)/Pbx and *homothorax* (*hth*)/Meis, which assist Hox proteins to form stable ternary DNA-protein complexes with much-enhanced specificity. This involves contacts with the conserved Hox Hexapeptide (HX) motif near the HD N-terminus, or alternatively, with the paralog-specific UBD-A motif detected in Ubx and Abdominal-A (Abd-A) proteins [15], [16]. Other TFs that can serve as positional Hox partners include the segment-polarity gene products Engrailed (En) and Sloppy paired, that collaborate with Ubx and Abd-A to repress abdominal expression of *Distal-less* [17]. Finally, specific a.a. residues in the HX motif, the HD and the linker separating them play a distinctive role in DNA target specificity, allowing one Hox HD region to select paralog-specific targets [18], [19].

Contrasting with our knowledge of collaborations involving Hox and partner TFs, virtually nothing is known of what transpires at the interface with the RNA Polymerase II (PolII) machinery itself to generate an appropriate transcriptional response. The lone evidence directly linking Hox TFs to the PolII machine comes from the observation that the *Drosophila* TFIID component BIP2 binds the Antp HX motif [20].

Another key component of the PolII machinery is the Mediator (MED) complex conserved from amoebae to man that serves as an interface between DNA-bound TFs and PolII. MED possesses a conserved, modular architecture characterized by the presence of head, middle, tail and optional CDK8 modules. Some of the 30 subunits composing MED appear to play a general structural role in the complex while others interact with DNA-bound TFs bridging them to PolII. Together, these subunits and the MED modules they form associate with PolII, TFs and chromatin to regulate PolII-dependent transcription [21]–[28].

Our analysis of a *Drosophila skuld/Med13* mutation isolated by dose-sensitive genetic interactions with homeotic *proboscipedia* (*pb*) and *Sex combs reduced* (*Scr*) genes led us to view MED as a Hox co-factor [29]. However, how MED might act with Hox TFs in developmental processes has not been explored. The work presented here pursues the hypothesis that Hox TFs modulate PolII activity through direct binding to one or more MED subunits. Starting from molecular assays, we identify Med19 as a subunit that binds to the homeodomain of representative Hox proteins through an animal-specific motif. Loss-of-function (lof) *Med19* mutations isolated in this work reveal that Med19 affects Hox developmental activity and target gene regulation. Taken together, our results provide the first molecular link between Hox TFs and the general transcription machinery, showing how Med19 can act as an embedded functional partner, or “co-factor”, that directly links DNA-bound Hox homeoproteins to the PolII machinery.

## Results

### Hox proteins directly bind Med19 via their homeodomain

**Figure 1:**
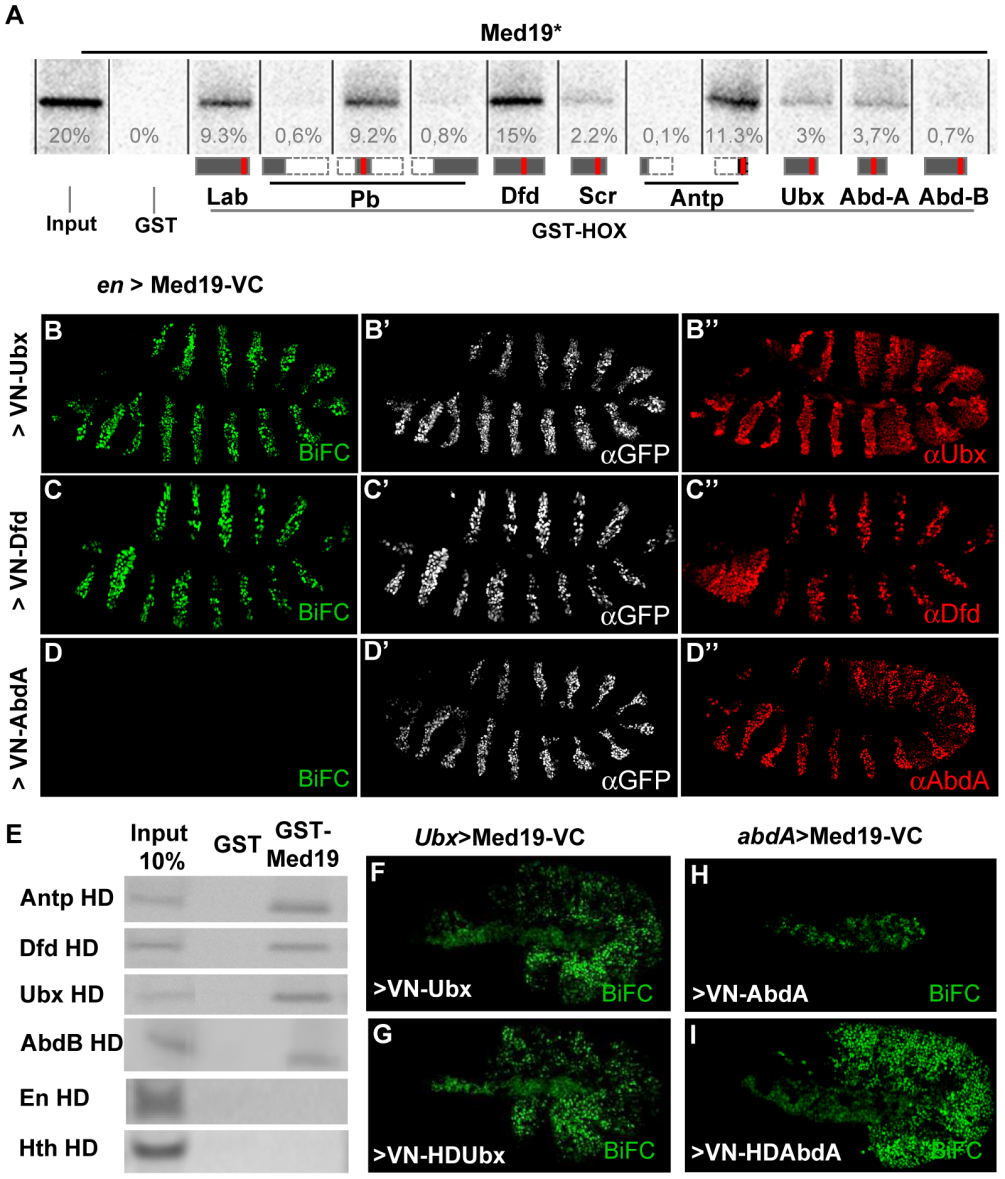
Hox proteins bind Med19 through their homeodomains *in vitro* and *in vivo*. (A) GST-pulldown binding assays of ^35^S-Med19 to immobilized GST-Hox fusions containing full length or protein fragments (below each lane: rectangles represent the entire protein; portions present in GST-Hox chimeric proteins are black, except the HD, represented in red). (B–D) BiFC assays were carried out co-expressing Med19-VC with VN-Ubx (B), VN-Dfd (C) or VN-AbdA (D), from UAS constructs under *engrailed*-Gal4 control (*en*>). Med19-VC accumulation, detected with antibody against the GFP C-terminal region, is similar in all tests (B′–D′). Gal4-driven Hox protein accumulation is comparable to endogenous, as detected with Ubx, Dfd and AbdA specific antibodies (B″–D″). Relative BiFC fluorescent signals were quantified as in [32]. VN-Ubx signal (B) and VN-Dfd (C) yielded serial rows of nuclear fluorescence; VN-AbdA (D) gave no detectable signal. (E) Direct homeodomain binding to Med19. Pulldowns with immobilized GST or GST-Med19 employed 70 aa-long ^35^S-labelled peptides centered on the HDs of Antp, Dfd, Ubx, AbdB, En and Hth. (F–I) Direct homeodomain binding to Med19 in BiFC assay. Co-expression of Med19-VC with VN-Ubx (F), or with its HD (VN-HDUbx; G), under *Ubx*-Gal4 control gives indistinguishable BiFC signals. Expression of Med19-VC under *abdA*-Gal4 control yielded no fluorescence with VN-AbdA (H) but gave a strong signal with VN-HDAbdA alone (I).

To search for MED subunits that contact Hox proteins directly, a Hox/MED binding matrix was established with the *in vitro* GST-pulldown assay. GST fusions of the eight *Drosophila* Hox proteins (full-length Labial (Lab), Deformed (Dfd), Scr, Ubx, Abd-A and Abdominal-B (Abd-B), or portions of Pb and Antp) were probed with ^35^S-labelled MED proteins in standard conditions for 11 MED subunits that are not required for cell viability in yeast and/or that are known to interact physically with TFs in mammalian cells (Materials and Methods). The Med19 subunit stood out, binding strongly to multiple GST-Hox proteins in this assay (mean binding, ≥5% of input in multiple tests, except for Abd-B, 0.8%; Figure 1A).

To provide an independent and *in vivo* test for direct Med19/Hox binding, we used the Bimolecular Fluorescence Complementation (BiFC) assay. Auto-fluorescence from the Venus variant of Green Fluorescent Protein (GFP), abolished by truncating Venus protein into N- and C-terminal portions (VN and VC), can be effectively reconstituted when VC and VN fragments are coupled to interacting protein partners that reunite them in cultured cells [30] or living organisms [31], [32]. BiFC tests made use of the Gal4/UAS system [33] to direct co-expression of chimeric VN-Hox and Med-VC proteins. The functional validation of VN-AbdA relative to AbdA has been described [32]. VN-Ubx and VN-Dfd were likewise validated by their gain-of-function (gof) transformations in embryos (Figure S1). Fused Med19-VC was validated by its capacity to rescue lethal *Med19* mutants (described below). UAS-directed co-expression of Med19-VC with VN-Ubx, VN-Dfd or VN-AbdA in embryos under *engrailed*-Gal4 control (*en*>Med19-VC+VN-Hox) resulted in serial stripes of clear nuclear fluorescent signal for VN-Ubx and VN-Dfd (Figure 1B–C). VN-AbdA was negative in this test (Figure 1D, but see Figure 1H, I below). Nevertheless, the concordant results from GST pulldown and BiFC tests indicate direct Med19 binding to Ubx and Dfd.

The observed direct Med19/Dfd or Ubx binding led us to ask whether these interactions utilize the homeodomain (HD) common to all Hox proteins. Consistent with this possibility, GST fusions containing the HX-HD regions of Pb (middle, a.a. 119–327) and Antp (C-ter, aa 279–378) bound Med19 at levels 15- to 50-fold superior to Pb N-ter (a.a. 1–158), Pb C-ter (a.a. 267–782) and Antp N-ter (a.a. 1–90) peptides (Figure 1A). On dissecting the HD-containing C-terminal regions of Ubx, GST-Med19 bound similarly to wild-type and HX-mutated Ubx [15], or to the linker-shortened C-terminal region of crustacean *Artemia salina* Ubx (Figure S2). Tests with the Antp C-ter peptide (a.a. 279–378) containing its HX, linker and HD, led to the same conclusion (not shown). Confirmation came from binding experiments using immobilized GST-Med19 and Antp, Ubx, Dfd and Abd-B HD peptides, where strong binding was observed for all four homeodomains despite marked divergence of their primary sequences (60% identity of Abd-B and Ubx HD) (Figure 1E). Contrary to the four Hox HD, neither Engrailed HD (43% identity with Ubx HD) nor the TALE class Hth HD (21% identity) bound detectably (Figure 1E). Since Med19 binds Dfd, Antp, Ubx and Abd-B HD but not those of En or Hth, we infer that it is specific and can discriminate among homeodomains.

In BiFC assays for Med19/HD association *in vivo*, comparable signals were observed on co-expressing Med19-VC with full-length VN-Ubx, or its HD alone (VN-HDUbx), in the normal *Ubx* expression domain (*Ubx*-Gal4; Figure 1F,G). Co-expressing VN-AbdA with Med19-VC under *abdA*-Gal4 did not give rise to fluorescence (Figure 1D, H). By contrast, AbdA HD (VN-HDAbdA) yielded a strong fluorescent signal (Figure 1I), confirming that the Hox HD is sufficient for direct Med19 binding. The differing responses obtained for full-length AbdA versus its HD raise the interesting possibility that AbdA sequences outside the HD limit its access to Med19 *in vivo*. Taken together, these biochemical and *in vivo* results indicate that the Hox HD is sufficient for direct binding to Med19.

### *Med19* participates in Hox organizing activity

**Figure 2:**
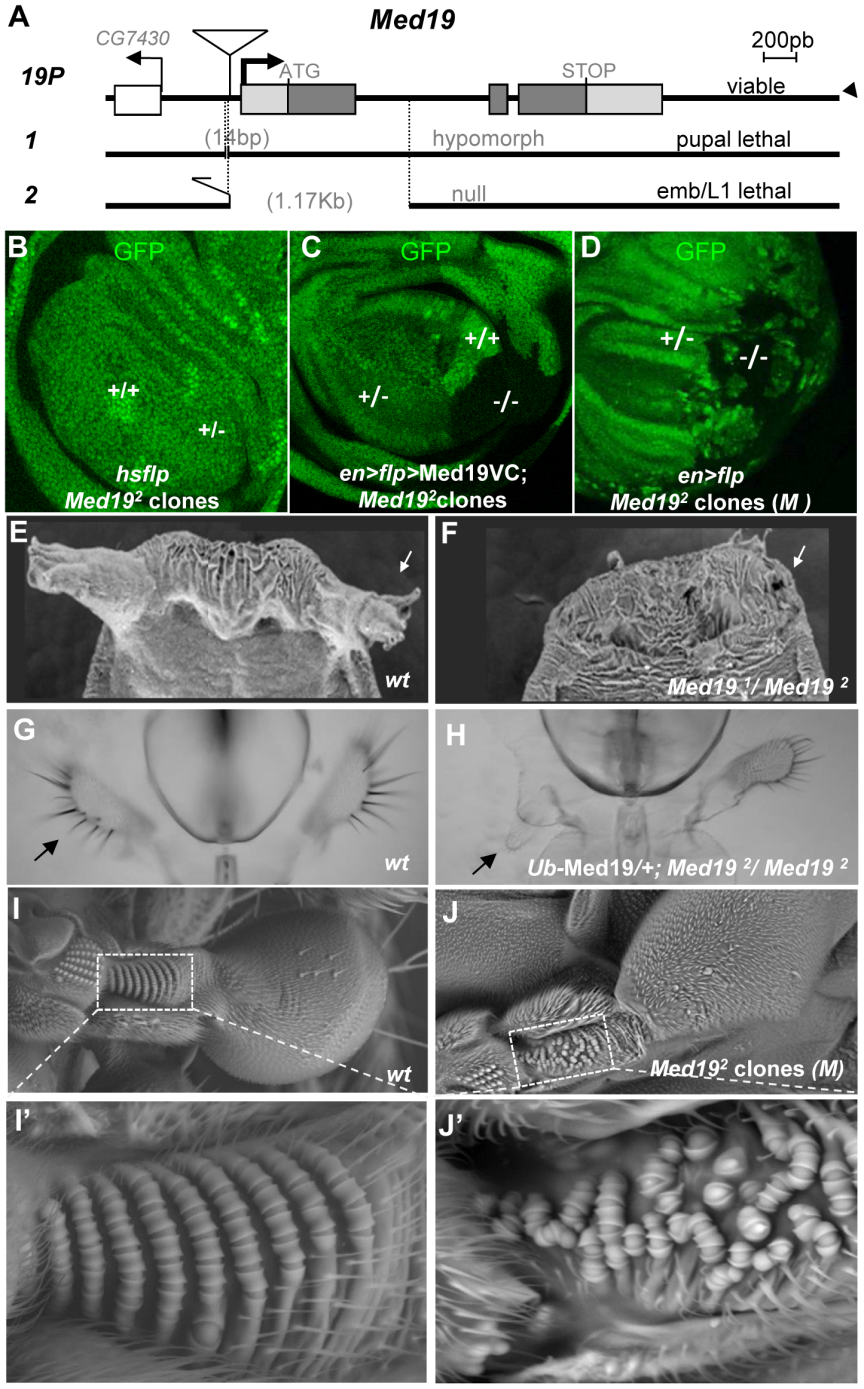
*Med19* mutations affect cell viability and Hox-related developmental processes. (A) Mutant alleles were generated by imprecise excision of a viable *P* element insertion 37 bp upstream of the putative *Med19* transcription initiation site (*19P*). *Med19^1^* is a pupal-lethal hypomorph with 14 bp deleted 5′ to the *Med19* transcription start. *Med19^2^* is an embryonic lethal amorph deleted for 1174 bp of DNA spanning exon 1 with its ATG initiation codon. (B–D) Clonal analyses of *Med19^2^*. (B) “Twin spot” analysis. Mitotic recombination induced in *hsp70*-Flp; *Med19^2^* FRT-2A/Ub-GFP FRT-2A larvae (30′ heat shock at 38°C) gave +/+ clones (intense green), but no −/− sister clones (GFP-) were observed. (C) Twin spot analysis in rescue conditions. The engrailed-Gal4 driver was used to simultaneously induce mitotic clones (UAS-Flp) and to direct expression of Med19-VC (UAS transgene). Homozygous *Med19^2/2^* (−/−) cells (lacking GFP) are now detected. (D) The *Med19* ^−^ condition is not intrinsically cell-lethal. In this wing imaginal disc (genotype, *en-*Gal4>UAS-Flp/+; *Med19^2^* FRT-2A/Ub-GFP *M^−^* FRT-2A), GFP- *Med19* ^−/−^ clones are observed. (E–J) *Med19* function is required for multiple Hox-related developmental processes. (E,F) *Med19* is required for eversion of anterior pupal spiracles. Normal anterior spiracles (E) are absent from *Med19^1^* ^/*2*^ hypomorphs (F). (G,H) One maxillary palp (G, arrow) is absent in a surviving Ub-Med19; *Med19^2/2^* hypomorph (H, arrow). (I,J) *Med19* is required for haltere-specific sensory organs. (I) Wild-type haltere, with zone of interest (dotted box) showing rows of pedicellar sensillae on the wild-type dorsal haltere (enlargement, I′). (J) Haltere harboring *Med19* ^−/−^ clones (genotype: *ap*>Flp; Ub-GFP *M^−^* FRT-2A/*Med19^2^* FRT-2A), with zone of interest in dotted box indicating disorganized sensory organ rows (J′).

Apart from yeast, no mutants of *Med19* have been described. To address *Med19* gene function *in vivo*, we employed imprecise excision of a viable *P* element insertion mutant, *Med19^P^* to generate two loss-of-function (lof) mutations, *Med19^1^* (a pupal-lethal hypomorphic allele harboring a 14 base pair (bp) upstream deletion), and *Med19^2^* (deleted for much of its protein coding sequence; see Figure 2A). Homozygotes for the presumptive null mutation *Med19^2^* die at the end of embryogenesis but do not show cuticular defects. To remove maternally contributed protein and/or mRNA [34] that might mask early requirements for Med19, we used the Dominant Female Sterile technique to generate mitotic germ-line clones. When clones were induced in females heterozygous for *Med19^2^* and for *ovo^D1^*, egg-laying was observed. Such embryos devoid of maternally contributed *Med19* product undertake development (as visualised by nuclear DAPI staining, Figure S3). Though the first nuclear divisions proceed normally (Figure S3, ≈1 hr), abnormalities are already visible in pre-cellular blastoderm (Figure S3, ≈2 hr), leading to massively disorganised cellular embryos that die soon after (Figure S3). We conclude that a major maternal contribution to embryonic *Med19* activity masks its zygotic roles.

As an alternative approach to examining *Med19* function in embryonic development, we made use of Med19-directed RNAi. Ubiquitous RNAi expression under *daughterless*-Gal4 control gave rise to cuticular defects in the spiracles and head of L2/L3 larvae (Figure S4) reminiscent of the embryonic consequences of Abd-B lof in the posterior spiracles, or of lab/Dfd/Scr lof in the head. However, the late appearance of these defects, their incomplete penetrance and variable expressivity made it difficult to ascertain a functional link to embryonic Hox activities.

We therefore decided to examine post-embryonic development, making use of partial loss-of-function combinations and of clonal analysis. *Med19^1^/Med19^2^* animals die as pupae, but adult viability is restored by ubiquitous transgenic expression of Med19 (Ub-Med19 or *arm*>Med19-VC; Figure S9). These results show that lethality is due solely to loss of *Med19* function. They also functionally validate the UAS-Med19-VC element used in BiFC assays. To better characterize the consequences of *Med19* loss-of-function, we employed FLP/FRT-mediated mitotic recombination [35] to generate clones of cells homozygous for *Med19^2^* (*−/−*). In “twin spot” experiments where −/− and +/+ cells arise from mitotic recombination in a single −/+ cell during mitosis, only +/+ cells were subsequently detected (Figure 2B). This cell lethality is due to loss of Med19, since expressing Med19-VC protein in mutant cells restores viability (Figure 2C); indeed, large *Med19^2^/Med19^2^* clones are observed even though Med19-VC accumulation is less than for wild-type protein in adjacent cells (Figure S5,A–A″). Strikingly, *Med19^2^/Med19^2^* cells also survived in the presence of a *Minute* (*M*) mutation [36] that slows growth of surrounding heterozygous *M^−/+^* cells (Figure 2D). Immune staining with anti-Med19 sera confirmed that *Med19^2^* is a protein-null mutation (Figure S5, B–B″). Thus the existence of these clones shows that Med19 is not strictly required for cell viability. The influence of cell environment on cell lethality suggests a role for Med19 in the control of cell competition.

If Hox/Med19 binding is functionally relevant to homeotic activity, Med19 mutants might provoke Hox-like phenocopies or modify Hox-induced homeotic defects. In light of the strong maternal contribution of Med19 present in embryos, we turned our attention to later developmental stages. Hypomorphic *Med19^1^/Med19^2^* animals, or *Med19^2^/Med19^2^* animals with low-level ubiquitous expression from the Ub-Med19 transgene, survive to the pupal stage and show a fully penetrant loss of anterior spiracles (Figure 2E, F). Rare adult Ub-Med19; *Med19^2^/Med19^2^* survivors showed defects including loss of maxillary palps (Figure 2G, H). Tissue-directed induction of *Med19^2^/Med19^2^* clones in the dorsal compartment of the haltere imaginal disc (*apterous*-Gal4>UAS-Flp) is associated with disorganization of the distinctive pedicellar sensillae in halteres [37], where these sensory organs are reduced in number and their well-ordered rows disrupted in adult halteres (Figure 2I–I′ vs. 2J–J′).

**Figure 3:**
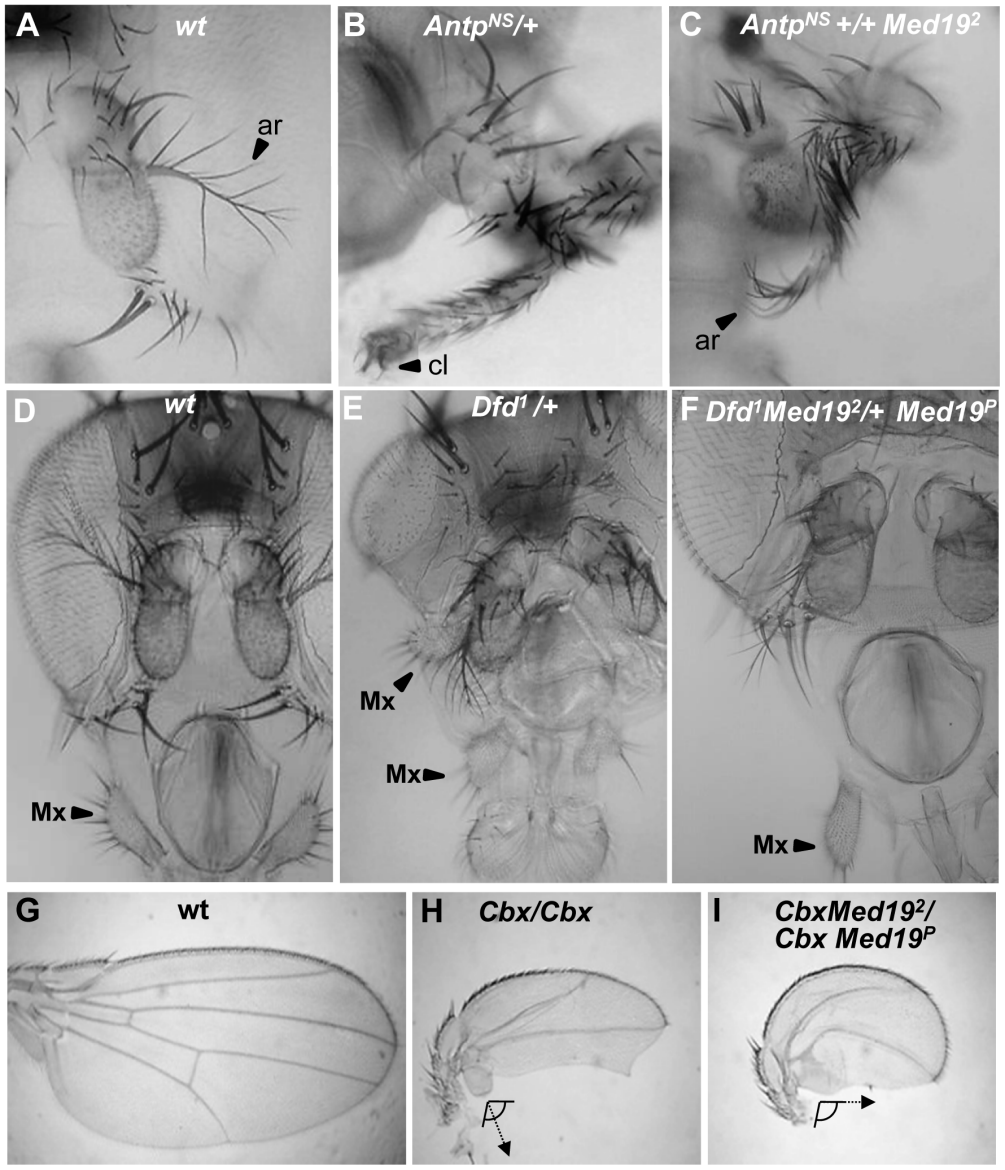
Synergistic interactions between *Med19* and Hox mutations. Dose-sensitivity for *Med19* was tested relative to Hox gain-of-function mutations of *Antp* (A–C), *Dfd* (D–F), and *Ubx* (G–I). (A) Wild-type antenna, with distal arista (ar) indicated by an arrowhead; (B) *Antp^Ns^*–directed transformation of antenna toward leg with distal claw (cl, arrowhead); (C) the transformation is attenuated in *Antp^Ns^*/*Med19^2^* trans-heterozygotes, as shown by the presence of a partial arista (ar, arrowhead). (D) Wild-type head, with the maxillary palp (Mx) indicated by arrowhead. (E) *Dfd^1^* provokes head defects including reduced eyes and the appearance of ectopic Mx (arrowhead), here positioned behind the antenna. (F) In *Dfd^1^*/*Med19^2^* heterozygotes (or here, *Dfd^1^Med19^2^*/+ *Med19^P^*), no ectopic Mx were observed. (G) Wild-type wing. (H) Homozygote for the *Ubx^Cbx1^* gof allele that expressed Ubx protein in the posterior compartment of the wing. Note the discrete hemi-haltere induced by Ubx, which is oriented at right-angles relative to the longitudinal wing axis. (I) In *Ubx^Cbx1^ Med19^2^*/*Ubx^Cbx1^ Med19^P^* wings, the posterior wing is no longer organized as a hemi-haltere, and the cellular trichomes are reoriented toward the long wing axis (arrow).

All the preceding defects (Figure 2E–J′) resemble Hox loss-of-function phenotypes: of *Antp* for the pupal anterior spiracles [38]; of *Dfd* for the adult maxillary palps [39], [40]; and of *Ubx* for haltere sensory organs [37]. We therefore asked whether *Med19* mutants can act as dose-sensitive modifiers of *Hox* activity in genetic interaction tests. For *Antp*, the fully penetrant spiracle loss observed in *Med19^1^/Med19^2^* pupae (Figure 2F) is absent from *Med19^−^* or *Antp^−^* heterozygotes but can be detected in *Med19^−^*/*Antp^−^* double heterozygotes (Figure S6). This synergistic interaction links *Med19* to normal *Antp* function. Conversely, ectopic expression from the gain-of-function (gof) *Antp^Ns^* allele directs a fully penetrant transformation of antenna toward leg (Figure 3A, B) that is partially suppressed in *Med19^2^* heterozygotes, as shown by the replacement of distal claws by antennal aristae (Figure 3C, Figure S6). Adult maxillary (Mx) palps (Figure 3D, arrowhead) require *Dfd* function in a territory abutting the antennal primordium of the eye-antennal imaginal disc [40], and the palp loss noted above (Figure 2H) suggested a link to *Dfd*. In interaction tests, palp loss provoked by *Dfd*-specific RNAi (*patched*>dsRNADfd) was enhanced in *Med19^2^* heterozygotes (Figure S6). Conversely, ectopic Mx organs observed in heterozygotes for the gof allele *Dfd^1^* (20% of adult heads) were fully suppressed in *Dfd^1^*/*Med19^2^* double heterozygotes (Figure 3E, F and Figure S6). Similarly, the transformation of the posterior wing (Figure 3G) to a hemi-haltere in *Ubx^Cbx1^* homozygotes (Figure 3H) was synergistically modified on reducing Med19 activity (Figure 3I). These dose-sensitive modifications of *Antp*, *Dfd* and *Ubx*-dependent phenotypes by *Med19* lof mutants support a functional Hox/Med19 link *in vivo*.

### Med19 is required for Ubx target gene activation

**Figure 4:**
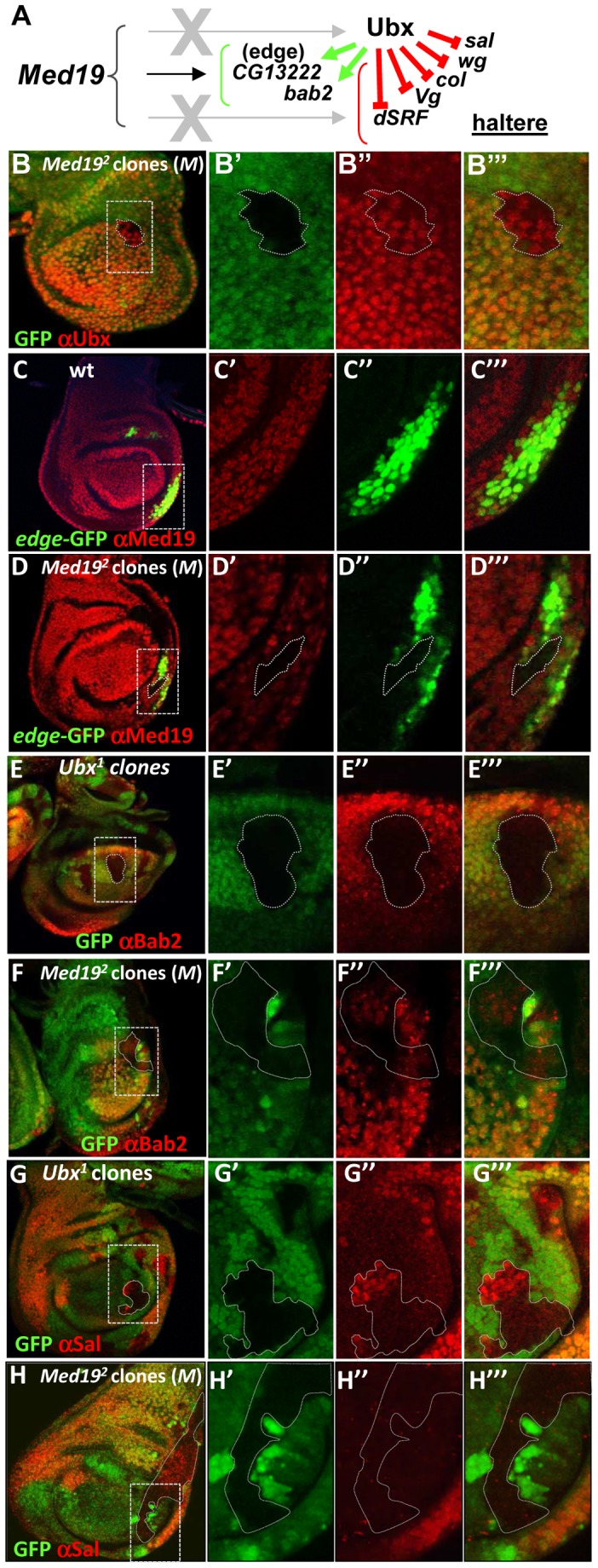
Med19 acts as a “co-factor” for Ubx-mediated gene activation. (A) Summary of Ubx target genes analyzed. Ubx can repress (red bars) or activate (green arrows) direct target genes. (B–H) *Med19* function in Ubx target gene expression. Images show whole haltere imaginal discs that are wild-type (C), or bear mitotic clones of *Med19^2^* in a *Minute* background (B, D, F, H) or of *Ubx^1^* (E,G). The other columns contain enlargements of boxed images in the first column, showing genotypic markers to identify clones (B′–H′); expression of gene of interest (B″–H″); and merged images (B′″–H′″). (B–B′″) Ubx protein (red) accumulates normally in a *Med19* ^−/−^ clone (GFP-). (C–C′″) *edge*-GFP reporter gene expression (green) is localized in a row of cells at the posterior border of the disc; ubiquitous expression of wild-type Med19 is revealed by anti-Med19 (red). (D–D′″) *edge*-GFP expression (green) is absent in a *Med19* ^−/−^ clone (absence of anti-Med19, red; circled) crossing the line of *edge*-expressing cells. (E–E′″) Bab2 protein (anti-Bab2, red) is absent from *Ubx^1/1^* cells (GFP-). (F–F′″) Bab2 expression (red) is cell-autonomously down-regulated in *Med19* ^−/−^ cells (GFP-). (G–G′″) *spalt* (*sal*) expression (anti-Sal, red) appears in centrally positioned cells of *Ubx^−/−^* clones (GFP-) in the haltere pouch. (H) Spalt (red) is not de-repressed in Med19 mutant cells (GFP-) positioned as for the *Ubx* clone (G′).

The intensive attention given to Ubx target gene regulation in the haltere imaginal disc [3]–[5], [41], [42] makes it an excellent paradigm for understanding Hox interplay with Med19 in a developmental program. We therefore examined the effect of removing *Med19* function on Ubx activity towards selected target genes in the haltere imaginal disc (Figure 4A). Clones of *−/−* cells induced in haltere discs in the presence of a *Minute* mutation showed Ubx levels comparable to their +/− neighbors (Figure 4B–B′″). The presence of normal nuclear Ubx signal in mutant cells after several mitotic divisions shows that the *Med19^−^* condition has not generally affected transcription. We next examined the effects of *Med19^−/−^* mitotic clones on several Ubx target genes in haltere development [41]. Ubx is known for its role in suppressing wing development, and acts to repress a number of prominent wing developmental genes in the haltere imaginal disc [41]. With the combined use of ectopic Hox expression and analysis of the transcriptome, additional Ubx targets have emerged. The first example of a Ubx-activated target was *CG13222*, which is regulated through an autonomous *cis*-regulatory region called “edge” for its expression in a band of cells along the posterior border of the haltere disc [42]. Expression of the *edge*-GFP reporter construct, that recapitulates Ubx-dependent *CG13222* expression [42] is cell-autonomously abolished in *Med19^−/−^* haltere clones (Figure 4C,D; identified with anti-Med19 sera). This shows that Med19 is required for activation of the direct Ubx target *CG13222* in the haltere disc. A second positively-regulated target identified in recent whole-genome analyses, *bric-à-brac2* (*bab2*), is induced by ectopic Ubx [5] and correlates with direct Ubx binding to regulatory DNA *in vivo* [3], [4]. Using mitotic recombination, we examined the expression of *bab2* in *Ubx^−/−^* haltere cells and found that Bab2 accumulation is cell-autonomously abolished (Figure 4E–E′″). On examining *bab2* expression with respect to *Med19* activity, Bab2 accumulation was cell-autonomously down-regulated in *Med19^−/−^* cells (Figure 4F–F′″). This shows that *Med19* activity is required in the haltere disc for normal Ubx-mediated activation of *bab2*. However, contrary to *edge*, residual low-level *bab2* expression is present in some *Med19* ^−^ cells (Figure 4F–F′″). The responses of these two regulatory sequences to *Med19* lof indicate that Med19 is required for Ubx target gene activation.

Several targets whose expression is repressed by Ubx were tested for a requirement for Med19 (Figure 4A). For example, the broad central band of *spalt* (*sal*) expression in the wing pouch is absent from Ubx-expressing cells of the haltere pouch. *sal* is de-repressed in *Ubx^−/−^* haltere disc cells [41], as shown by the appearance of Sal protein in *Ubx* mutant cells (Figure 4G–G′″). By contrast, no new expression of Sal is detected in equivalently placed *Med19^−/−^* clones (Figure 4H–H′″). De-repression was likewise not observed for other tested Ubx-repressed targets (not shown), lending molecular support to the interpretation that Med19 is not involved in Ubx repressive activities. While further examples will be required to determine whether this illustrates a general property of Med19 action in transcriptional regulation, these results suggest that Med19 collaboration with Ubx is limited to gene activation.

### Med19 binds the Hox homeodomain through a conserved motif

**Figure 5:**
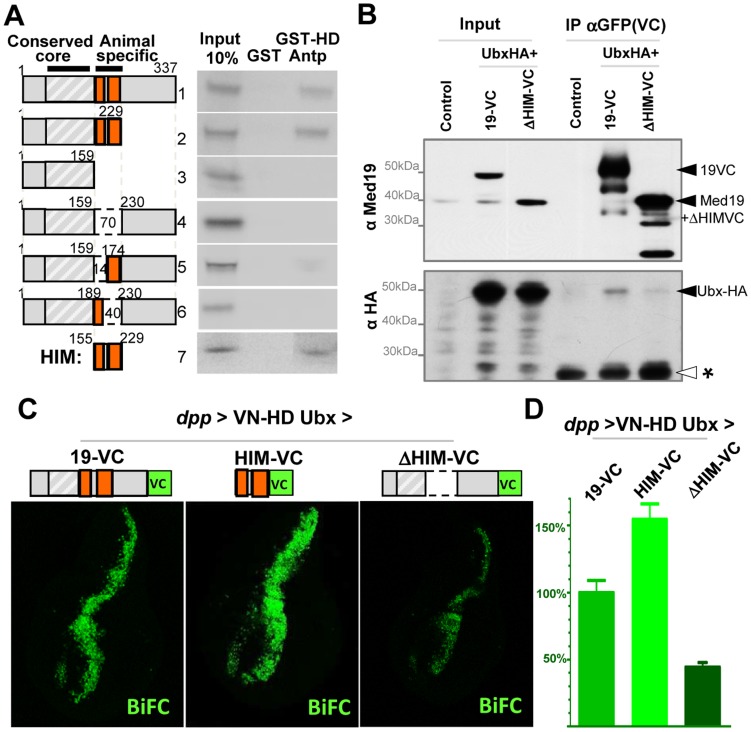
Direct HD binding through a conserved 70 a.a. Med19 homeodomain-interacting motif (HIM). (A) HD binding involves a 70 a.a. region of Med19. ^35^S-labelled full-length (construct #1) or deleted versions (#2–7) of Med19 were used to probe immobilized GST or GST-HDAntp. Proteins containing a.a. 159–229 bound GST-HD (#1, 2, 7). Deleting the entire interval (#4) or of a 40 a.a. interval from 190–229 (#6) abolished binding. Deleting the N-terminal 14 aa of this region (160–173) resulted in reduced binding (#5). The 70 a.a. HIM peptide (160–229) bound GST-HD (#7). (B) Co-immunoprecipitations. Transfected S2 cells contained pActin-Gal4 driver with pUAS-Ubx-HA and either pUAS-Med19-VC or pUAS-Med19ΔHIM-VC. Negative controls were cells transfected with pAct5C-V5. Inputs represent 2% of extracts used for the IP. Cell extracts were immunoprecipitated with mouse anti-GFP sera that recognises the VC tag, then analysed by Western blots. In the upper portion, bands were revealed with guinea pig anti-Med19, while in the lower portion, a duplicate blot was stained using rabbit anti-HA sera. Solid arrowheads indicate identified proteins of interest and “*”, a non-specific signal serving as an internal loading control. As the HIM motif and VC tag are of equal size, endogenous Med19 and ΔHIM-VC migrate at the same position. (C,D) BiFC test, co-expressing VN-HDUbx with Med19-VC, Med19ΔHIM-VC or HIM-VC in the wing imaginal disc from UAS constructs under *dpp*-Gal4 control. (C) The BiFC signal observed for VN-HDUbx with Med19-VC was higher for HIM-VC while it was reduced to background levels with Med19ΔHIM-VC. (D) Quantification of BiFC fluorescent signals.

Binding of Hox proteins to Med19 specifically involves their conserved homeodomain. To identify Med19 sequences involved in HD binding, we used GST-pulldown to test full-length or deleted versions of Med19 with GST-HDAntp. Binding was retained on truncating the terminal regions of Med19 (Figure 5A, constructs 1–2), but was abolished on deleting an internal 70 aa region (Figure 5A, constructs 3–4). Smaller deletions confirmed that this region contains sequences required for full binding (Figure 5A, constructs 5–6). This 70 a.a. Med19 peptide is not only required but also proved sufficient to bind the HD (Figure 5A, construct 7), leading us to call it Homeodomain Interacting Motif (HIM).

The HIM interval of Med19 was then compared with Med19 orthologs from a spectrum of eukaryotes. Sequence alignments reveal a lysine/arginine-rich sequence that is strongly conserved in Med19 orthologs from six vertebrate or insect species (Figure S7). This striking conservation suggests that the contribution of HIM to Med19 function is subject to strong selective pressure.

Med19 is required for Ubx-mediated activation of specific target genes *in vivo*, and directly binds Hox HDs *in vitro* through its conserved HIM motif. This suggested that Ubx function passes through Med19, potentially via its HIM sequence. We therefore sought evidence for Med19/Hox binding in cellulo. Cultured *Drosophila* cells expressing UAS-Med19-VC or UAS-Med19ΔHIM-VC were used for co-immunoprecipitations with anti-GFP (VC). As shown in the Western blot of Figure S8, the three endogenous Med1 isoforms were associated with both Med19-VC and Med19ΔHIM-VC. This is consistent with the incorporation of full-length and HIM-deleted forms into the MED complex.

We next co-expressed these proteins with Ubx-HA and tested for their association in cellulo. As seen in Figure 5B, Ubx-HA co-precipitates with Med19-VC, indicating the association of Ubx transcription factor with Med19. By contrast, less Ubx-HA was detected on co-precipitating with Med19ΔHIM-VC (relative to a non-specific band that serves as a de facto internal loading control, * in Figure 5B). These results indicate that the HIM domain contributes to Ubx-Med19 interaction.

To further investigate the contribution of the HIM domain to HD binding *in vivo*, we generated transgenic lines containing the same UAS-Med19-VC, -Med19ΔHIM-VC and -HIM-VC constructs used above, and tested each protein's ability to bind to VN-HDUbx in the BiFC assay. In control experiments, Med19-VC, HIM-VC and Med19ΔHIM-VC accumulated at comparable levels in wing imaginal discs (Figure S8). Med19-VC and HIM-VC are fully nuclear. The Med19ΔHIM-VC protein (lacking the highly basic HIM element) is seen to accumulate in both the cytoplasm and the nucleus (Figure S8). This indicates that the HIM element contributes to nuclear localisation, together with other Med19 sequences. As noted in embryos (Figure 1G), co-expressing Med19-VC with VN-HDUbx under *dpp*-Gal4 control in wing imaginal discs resulted in clear fluorescent signal (Figure 5C). When HIM-VC was tested for its ability to interact with the Ubx HD, it gave rise to a fluorescent signal stronger than for intact Med19; by contrast, the Med19ΔHIM-VC fusion yielded only a background-level signal with VN-HDUbx (Figure 5C). Taken together, these results of biochemical and BiFC experiments indicate that Med19 HIM is necessary and sufficient for full HD binding.

### HIM is required for Ubx target gene activation

While Med19 bereft of its conserved HIM element can be incorporated into MED, as shown above, its functional requirements *in vivo* remained an open question. Accordingly, we tested whether *Drosophila* HIM is relevant to Med19 developmental functions. In a genetic rescue test, ubiquitous Med19-VC expression restored adult viability to pupal-lethal *Med19* ^1^ */Med19* ^2^ hypomorphs, whereas Med19ΔHIM-VC did not. Med19ΔHIM-VC also showed a reduced aptitude to rescue pupal spiracles, adult maxillary palps and haltere sensillae compared with Med19-VC (Figure S6). These results indicate a requirement for Med19 HIM in several Hox-dependent developmental processes.

**Figure 6:**
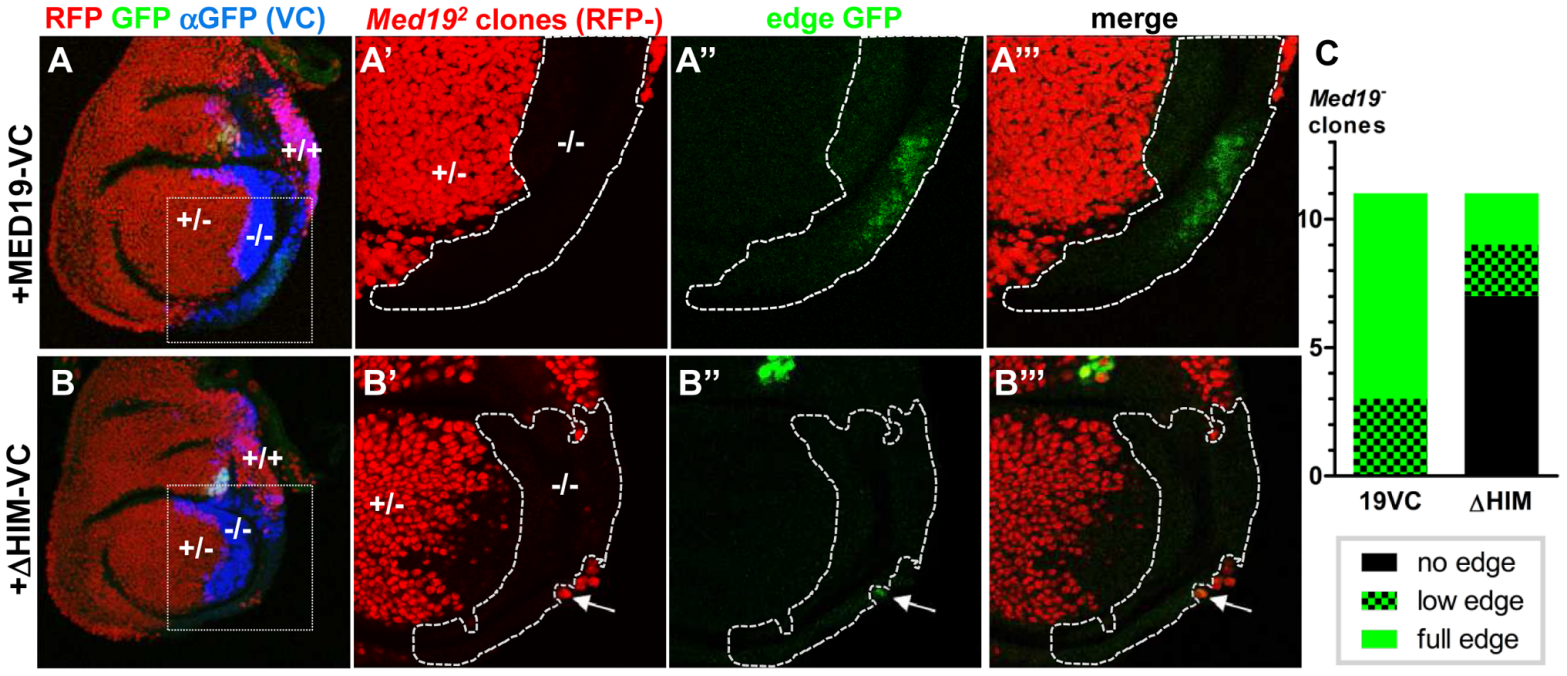
Med19 HIM is required for Ubx target gene activation, but not for cell proliferation/survival. Med19-VC (A) or Med19ΔHIM-VC (B) proteins were expressed (UAS constructs, *en*-Gal4) in posterior haltere imaginal discs harboring *Med19* mutant clones. Med19-VC or Med19ΔHIM-VC were detected with antisera directed against C-terminal GFP (αVC, blue). (A–A′″, B–B′″) *Med19* clones were identified using a ubiquitous RFP marker: −/− (no red), +/− (red), +/+ (intense red). Activation of the Ubx target *edge*-GFP at the posterior haltere edge was visualized by GFP (green). Regions containing *Med19^−^* ^/−^ clones of interest are enlarged (A′–A′″, B′–B′″). (A–A′″) Med19-VC restored expression of *edge*-GFP. (B–B′″) Med19ΔHIM-VC failed to rescue *edge*-GFP activation here. GFP-expression here is limited to a single wild-type cell that abuts the −/− clone (B′, B″, arrow). (C) Three levels of *edge*-GFP expression could be discerned: normal, present but reduced, or none. All correctly positioned −/− clones with Med19-VC showed GFP expression (11 of 11) of which 9/11 were normal. Most clones possessing Med19ΔHIM-VC showed no GFP (7 of 11), and only two of 11 clones showed normal expression.

We therefore sought to test the influence of the Med19 HIM peptide on Ubx-dependent transcriptional activation of the direct target *CG13222/edge*. To this end, (i) FLP/FRT-mediated mitotic recombination was used to generate cells devoid of wild-type protein, while (ii) UAS/Gal4-directed expression supplied normal or HIM-deleted Med19-VC, and (iii) the *edge*-GFP reporter was employed to assess Ubx-mediated activation of *CG13222*. *En*-Gal4-directed UAS-Flp expression in the posterior haltere disc compartment served to induce mitotic recombination there, while *en*-Gal4 simultaneously directed expression of Med19-VC or Med19ΔHIM-VC in the posterior compartment (Figure 6A–B, stained with anti-VC, blue).

These twin-spot experiments provided two important observations. Firstly, large −/− clones (RFP-) were observed not only in Med19-VC but also in Med19ΔHIM-VC expressing discs (Figure 6A′, B′). The existence of −/− clones is in marked contrast with their complete absence in Figure 2B. This shows that both Med19-VC and Med19ΔHIM-VC restore cell viability. We conclude that HIM is not necessary for cell viability. Further, it indicates that not only are both forms of Med19-VC incorporated into MED, but they are functional there. Secondly, Med19-VC and Med19ΔHIM-VC differed markedly in their capacities to ensure activation of the Ubx target gene *CG13222*. Reporter expression was observed within all appropriately positioned clones of −/− cells expressing Med19-VC (11 of 11 clones; Figure 6A″, C). By contrast, *edge*-GFP expression was entirely absent from most clones expressing Med19ΔHIM-VC (7 of 11 clones; Figure 6B″, C). The existence of HIM-independent cell proliferation/survival shows that this cellular function of Med19 can be uncoupled from its Hox-related role. These results also provide clear functional evidence that Ubx-dependent activation of its *edge*-GFP target requires HIM-endowed Med19.

## Discussion

### Med19, a MED regulatory subunit that binds Hox transcription factors

Hox homeodomain proteins are well-known for their roles in the control of transcription during development. Further, much is known about the composition and action of the PolII transcription machine. However, virtually nothing is known of how the information of DNA-bound Hox factors is conveyed to PolII in gene transcription. The *Drosophila Ultrabithorax-like* mutant affecting the large subunit of RNA PolII provokes phenotypes reminiscent of *Ubx* mutants [43], but the molecular basis of this remains unknown. The lone direct evidence linking Hox TFs to the PolII machine is binding of the Antp HX motif to the TFIID component BIP2 [20]. Here, we undertook to identify physical and functional links between *Drosophila* Hox developmental TFs and the MED transcription complex. Our results unveil a novel aspect of the evolutionary Hox gene success story, extending the large repertory of proteins able to interact with the HD [44] to include the *Drosophila* MED subunit Med19. HD binding to Med19 via the conserved HIM suggests this subunit is an ancient Hox collaborator. Accordingly, our loss-of-function mutants reveal that Med19 contributes to normal Hox developmental function and does so at least in part via its HIM element. Thus this analysis reveals a previously unsuspected importance for Med19 in Hox-affiliated developmental functions.

**Figure 7:**
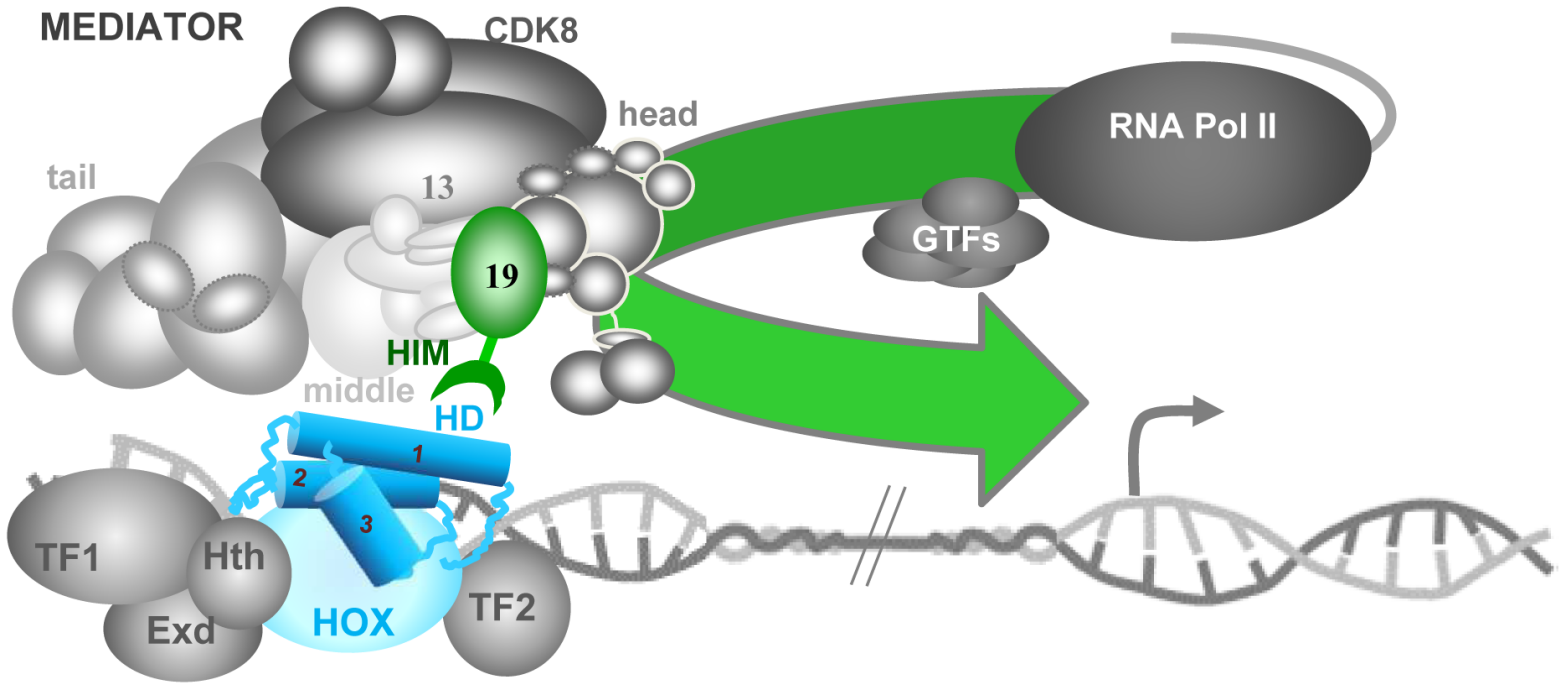
Model for the role of Med19 at the interface of Hox and MED. The Mediator complex, composed of four modules – tail, middle, head and CDK8 –, binds physically to PolII, principally through its head module. Hox transcription factors (HD in blue, its three α-helices indicated as cylinders) bind to regulatory DNA sequences distant from the transcription start site (grey arrow), together with unknown numbers of other TFs (here, Hox co-factors Exd and Hth plus cell-specific factors TF1 and TF2). We propose that the DNA-bound Hox homeodomain serves to recruit MED directly through Med19 HIM (green hook). This Hox-MED association then permits the general PolII transcription machinery (PolII+GTF) to be recruited to the Hox target promoter. This link to a MED subunit situated at the interface of the head, middle and CDK8 modules could modify overall MED conformation, favoring additional contacts between the TF complex and MED that modulate transcriptional activity.

A fundamental property of the modular MED complex is its great flexibility that allows it to wrap around PolII and to change form substantially in response to contact with specific TFs [45]. Recent work in the yeast *S. cerevisiae* places Med19 at the interfaces of the head, middle and CDK8 kinase modules [46], [47]. Med19 is thus well-positioned to play a pivotal regulatory role in governing MED conformation (Figure 7). Our results raise the intriguing possibility that MED structural regulation and physical contacts with DNA-bound TFs can pass through the same subunit. In agreement with this idea, recent work identified direct binding between mouse Med19 (and Med26) and RE1 Silencing Transcription Factor (REST) [48]. This binding involves a 460 a.a. region of REST encompassing its DNA-binding Zn fingers [48]. The present work goes further, in identifying a direct link between the conserved Hox homeodomain and Med19 HIM (Figure 7) that is, to our knowledge, the first instance for a direct, functionally relevant contact of MED with a DNA-binding motif rather than an activation domain.

### Hox-independent Med19 roles in cell proliferation/survival

*Med19* contributes to developmental processes with *Antp* (spiracle eversion), *Dfd* (Mx palp), and *Ubx* (haltere differentiation). Other phenotypes identified with our mutants indicate further, non-Hox related roles for Med19. As shown here, complete loss of *Med19* function leads to cell lethality that can be conditionally alleviated when surrounded by weakened, *Minute* mutation-bearing cells. These observations, that uncouple HIM-dependent functions from the role of Med19 in cell survival/proliferation (Figure 6B), are compatible with reports correlating over-expression of human Med19/Lung Cancer Metastasis-Related Protein 1 (LCMR1) in lung cancer cells with clinical outcome [49]. Further, RNAi-mediated knock-down of Med19 in cultured human tumor cells can reduce proliferation, and tumorigenicity when injected into nude mice [50]–[58]. A recent whole-genome, RNAi-based screen identified Med19 as an important element of Androgen Receptor activity in prostate cancer cells where gene expression levels also correlated with clinical outcome [59]. It will be of clear interest to examine how, and with what partners, Med19 carries out its roles in cell proliferation/survival.

### Transcriptional activation versus repression

The role played by mammalian Med19 and Med26 in binding the REST TF, involved in inhibiting neuronal gene expression in non-neuronal cells [48], [60], provides an instance of repressive Med19 regulatory function. We found that Med19 activity is required in the *Drosophila* haltere disc for transcriptional activation of *CG13222*/*edge* and *bab2*, but is dispensable for Ubx-mediated repression of five negatively-regulated target genes (Figure 4). Ubx can choose to activate or it can repress, at least in part through an identified repression domain at the C-terminus just outside its homeodomain [61]. Conversely Med19, which binds the Ubx homeodomain, appears to have much to do with activation.

Concerning the mechanisms of Ubx-mediated repression, one illuminating example comes from analyses of regulated embryonic *Distal-less* expression [17]. Ubx can associate combinatorially with Exd and Hth, plus the spatially restricted co-factors Engrailed or Sloppy-paired in repressing *Distal-less* [17]. Engrailed in turn is able to recruit Groucho co-repressor [62], suggesting that localized repression involves DNA-bound Ubx/Exd/Hth/Engrailed, plus Engrailed-bound Groucho. Groucho has been proposed to function as a co-repressor that actively associates with regulatory proteins and organizes chromatin to block transcription. Wong and Struhl [63] demonstrated that the yeast Groucho homolog Tup1 interacts with DNA-binding factors to mask their activation domains, thereby preventing recruitment of co-activators (including MED) necessary for activated transcription. The number of targets remains too small to be sure Med19 is consecrated to activation. Nonetheless, it will be of interest to determine whether Groucho can play a role in blocking MED/Ubx interactions that could provide an economical means for distinguishing gene activation from repression.

### The Hox-MED interface in evolution and development

The conserved Hox proteins and the gene complexes that encode them are well-known and widely used to study development and evolution. As to the evolutionary conservation of the Mediator transcription complex, the presence of MED constituents in far-flung eukaryotic species from unicellular parasites to humans [21] indicates that this complex existed well before the emergence of the modern animal Hox protein complexes. The DNA-binding domains are often the most conserved elements of TF primary sequence, and in the case of the Hox HD, recent forays into “synthetic biology” agree that this was the functional heart of the ancestral proto-Hox proteins [64]–[66]. Indeed, Scr, Antp and Ubx mini-Hox peptides containing HX, linker and HD motifs behave to a good approximation like the full-length forms, directing appropriate gene activation and repression resulting in genetic transformations [64]–[66]. Our results showing direct HD binding to Med19 HIM, and thus access to the PolII machinery, allow the activity of these mini-Hox proteins to be rationalized. We surmise that at the time when the Hox HD emerged to become a major developmental transcription player, its capacity to connect with MED through specific existing sequences was a prerequisite for functional success. One expected consequence of this presumed initial encounter with Med19 – a selective pressure on both partners and subsequent refinement of binding sequences – is in agreement with the well-known conservation of Hox homeodomains, and with the observed conservation of the newly-identified HIM element in Hox-containing eumetazoans. We imagine that subsequent evolution over the several hundred million years separating flies and mammals will have allowed this initial contact to be consolidated through subsequent binding to other MED subunits, ensuring versatile but reliable interactions at the MED-TF interface (Figure 7).

### A functional Hox-MED interface

Hox homeodomain proteins are traditionally referred to as selector or “master” genes that determine developmental transcription programs. The low sequence specificity of Hox HD transcription factors is enhanced by their joint action with other TFs, of which prominent examples, the TALE homeodomain proteins Extradenticle/Pbx and Homothorax/Meis are considered to be Hox co-factors. However, a Hox TF in the company of Exd and Hth could still not be expected to shoulder all the regulatory tasks necessary to make a segment with all the coordinated cell-types it is made up of, and collaboration with cell-type specific TFs appears to be requisite. A useful alternative conception visualizes Hox proteins not as “master-selectors” that act with co-factors, but as highly versatile co-factors in their own right that can act with diverse cell-specific identity factors to generate the cell types of a functional segment [67]. We envisage a model where a Hox protein would be central to assembling cell-specific transcription factors into TF complexes that interface with MED (Figure 7).

Such Hox-anchored TF complexes could make use of selective HD binding to Med19 as a beach-head for more extensive access to MED, such that loss of the Hox protein would incapacitate the complex: in the case of *Ubx* ^−^ cells, inactivating *bab2* or de-repressing *sal*. Accordingly, three observations suggest that binding of Hox-centered TF complexes involves additional MED subunits surrounding Med19 (Figure 7) : (i) *bab2* target gene expression is entirely lost in *Ubx*-deficient cells but can persist in some *Med19* ^−^ cells; (ii) *edge*-GFP in *Med19* ^−^ cells expressing Med19ΔHIM-VC was not altogether refractory to Ubx-activated *edge*-GFP expression (Figure 6); and (iii) Med19ΔHIM-VC is not entirely impaired for Ubx binding, as seen in co-immunoprecipitations (Figure 5). Thus Hox protein input conveyed through Med19-HIM at the head-middle-Cdk8 module hinge might provide an economical contribution toward organizing TF complexes that influence overall MED conformation [45] and hence transcriptional output. Decoding how the information-rich MED interface including Med19 accomplishes this will be an important part of understanding transcriptional specificity in evolution, development and pathology.

## Materials and Methods

### Ethics statement

Our work using *Drosophila* was performed in conditions in conformity with French and international standards.

### GST pulldowns

Culture and preparation of GST-fusion proteins, preparation of ^35^S protein probes, and pulldowns were carried out essentially as described in [68]. Chimeric GST-Hox constructs fused GST to Hox cDNAs. Full-length Hox fusions were used for Labial, Deformed, Sex combs reduced, Ultrabithorax, Abdominal-A and Abdominal-B. Fragments of Pb and Antp were present in the fusion proteins: Pb1 (N-ter, a.a. 1–158), Pb2 (middle with HD, a.a. 119–327) and Pb3 (C-ter, a.a. 267–782). For Antp, two GST fusions were used: Antp1 (N-ter, 1–90) and Antp4 (C-ter with HD, 279–378). Eleven MED putative surface subunits [21] could be expressed at useable levels in coupled *in vitro* transcription/translation reactions: Med1/Trap220, Med2/Med29/Ix, Med6, Med12/Kto, Med13/Skd, Med15/Arc105, Med19, Med25, Med30, Cdk8 and CycC.

### Co-immunoprecipitations

Cultured *Drosophila* S2 cells were transfected using FuGENE HD transfection reagent (Roche) with pActin-V5 (negative control; pActin-GAL4 driver with either pUAS-Med19-VC or pUAS-Med19ΔHIM-VC (MED co-IP); or adding pUAS-Ubx-HA (Med19-Ubx co-IP). 10^7^ cells were transfected with driver plasmid plus the UAS responder plasmid(s). After 72 hr, cells were harvested by scraping and pooled, collected by centrifuging then washed with 1x PBS. All subsequent steps until Western blotting were carried out at 4°C. Cell pellets were resuspended in IP buffer (50 mM TrisHCl, pH = 8, 150 mM NaCl, 0.5% NP40, 1 mM EGTA and Roche complete protease inhibitor cocktail), lysed by four-fold passage through a 27G needle, then centrifuged for 10 min at 14,500 rpm. Immunoprecipitation from 1.5 mg of total protein extract (5 µg/µl in IP buffer) was performed with mouse anti-GFP (ROCHE 4 µg/IP), with gentle agitation overnight. 15 µl of G-protein-coupled Sepharose beads (SIGMA, P3296) were added, then gently agitated for 2 hr. The non-bound fraction was discarded. Beads were washed 4 times with fresh IP buffer, taken up in 2X Laemmli buffer containing DTT and SDS, heated to 95°C, and centrifuged. Supernatants were then submitted to polyacrylamide gel electrophoresis. Med1 and Med19 were revealed using polyclonal sera from guinea-pig (diluted 1∶500), while Ubx-HA was detected with rabbit anti-HA (SIGMA) diluted 1∶1000.

### Bimolecular Fluorescence Complementation (BiFC)

Constructions corresponding to UAS-VN-Ubx, UAS-VN-AbdA and UAS-VN-HDAbdA transgenic lines are described in [32]. UAS-VN-Dfd, UAS-VN-HDUbx: Dfd and Ubx HD sequences were cloned into XhoI-XbaI sites downstream of the Venus VN fragment into the pUAST or pUASTattB plasmids described in [32]. UAS-Med19-VC, UAS-HIM-VC: Full-length Med19 coding sequences, or the internal HIM sequence generated from Med19 cDNA by PCR, were introduced as EcoRI-XhoI fragments to replace Hth coding sequences of pUaHth-VC [32]. For UAS-Med19ΔHIM-VC, internally deleted Med19 was generated from the full-length construct by double PCR, using the overlap extension method. The PCR-derived internal deletion product was cleaved by RsrII and XhoI, then cloned in place of the equivalent fragment of UAS-Med19-VC. All constructs were sequence-verified before fly transformation. Transgenic lines were established by classical P-element mediated germ line transformation or by site-specific integration using the ΦC-31 integrase. Embryos were analysed as described in [32]. For BiFC in imaginal discs, late third-instar larvae of appropriate genotypes were cultured in parallel in the same environmental conditions of temperature and larval density, then were dissected at the same time and fixed in the same solution (20′; 4% para-formaldehyde, 0.5M EGTA, 1X PBS). Wing and haltere imaginal discs were dissected and mounted in Vectashield (Vector Labs). Image acquisition was performed on a Leica SP5 using the same laser excitation, brightness/contrast and z settings. Confocal projections from at least 10 distinct wing discs per genotype were analyzed with ImageJ software.

### Fly strains

Stocks and crosses were maintained at 25°C on standard yeast-agar-cornmeal medium. Mutant stocks harboring *Antp^Ns^*, *Dfd^1^*, *Ubx^Cbx1^* and *Df(3L)BSC8* were from the Bloomington *Drosophila* Stock Collection. *Antp^Ns^* ^+Rc3^ was provided by R. Mann. *Edge*-GFP and *vgQ*-lacZ originate from the S. Carroll lab. The Ub-Med19 transgenic line expressing full-length Med19 cDNA under *ubi73* control is a homozygous-viable insertion on the X chromosome at attP site ZH 2A. These transgenic elements carry the visible marker mini-*white*. UAS-RNAi against Dfd is from a non-directed insertion on chromosome 2 (Vienna *Drosophila* Research Collection stock 50110). UAS-RNAi lines against Med19 are stocks 27559 and 33710 from the Bloomington collection. Gal4-expressing driver lines used were: *dpp*-Gal4 (imaginal disc-specific, AP boundary, anterior compartment), *arm*-Gal4 (ubiquitous), *ptc*-Gal4 (AP boundary, anterior compartment), *en*-Gal4 (posterior compartment), *ap*-Gal4 (dorsal compartment of wing and haltere discs), *Ubx*-Gal4 and *abdA*-Gal4 (abdominal expression under Ubx or abdA control, respectively).

### Generation of *Med19* mutants

Loss-of-function *Med19* alleles were generated by imprecise excision, mobilizing the viable P{EPgy2}EY16159 insertion marked with mini-*w*+ (see Flybase). Among 154 white-eyed candidates, two *Med19^1^* and *Med19^2^* (described in Figure 2) were hemizygous-lethal with *Df(3L)BSC8*.

### Rescue experiments

The following stocks were employed for rescue tests:

Ub-Med19; *Med19^2^*/TM6B, *Hu Tb*;

*Med19^1^*/TM6B, *Hu Tb*;

*Med19^2^/*TM6B, *Hu Tb*;

*Arm*-Gal4; *Med19^1^*/TM6B, *Hu Tb*;

UAS-Med19-VC; *Med19^2^*/TM6B, *Hu Tb*;

UAS-Med19ΔHIM-VC; *Med19^2^*/TM6B, *Hu Tb*.

### Clonal analyses

Mitotic clones were induced by Flp recombinase expressed from a hsp70-Flp transgene on heat induction (30′ at 38°C), or from a UAS-Flp element under Gal4 control as indicated above (*en*>Flp, *ap*>Flp). Clones were generated and identified in marked progeny from crosses using the following stocks:

*Med19^2^* FRT-2A/TM6B, *Hu Tb*

*hsp70*-Flp; Ub-GFP FRT-2A/TM6B, *Hu Tb*

Ub-Med19; Ub-GFP FRT-2A/TM6B, *Hu Tb*

UAS-Med19-VC; Ub-GFP FRT-2A/TM6B, *Hu Tb*

*en>*Flp; *Med19^2^* FRT-2A/TM6B, *Hu Tb*

Ub-GFP *M* FRT-2A/TM6B, *Hu Tb*

*ap>*Flp; *Med19^2^* FRT-2A/TM6B, *Hu Tb*

*hsp70*-Flp; Ub-GFP *M* FRT-2A/TM6B, *Hu Tb*

*edge-*GFP/CyO, *Cy; M* FRT-2A/TM6B, *Hu Tb*

*ap>*Flp; FRT-82B Ub-GFP/TM6B, *Hu Tb*

FRT-82B *Ki pb^5^ p^p^ Ubx^1^ e*/TM6B, *Hu Tb*

*en>*Flp; FRT-82B Ub-GFP/TM6B, *Hu Tb*

*edge-*GFP, UAS-Med19-VC/CyO, *Cy*; His2Av-mRFP1 FRT-2A/TM6B, *Hu Tb*

*edge-*GFP, UAS-Med19ΔHIM-VC/CyO, *Cy*; His2Av-mRFP1 FRT-2A/TM6B, *Hu Tb*

### Germ-line clones

After crossing *y w hsp70*-Flp; *Med19^2^* FRT-2A/TM6B, *Hu Tb* females with *w*; P[*ovo^D1^*] FRT-2A/TM3, *Sb* males, progeny at L3/early pupal stages were subjected to heat shocks (1 hr, 37°C) on two successive days. Resulting *y w hsp70*-Flp/*w*; *Med19^2^* FRT-2A/P[*ovo^D1^*] FRT-2A adult females were crossed with *Med19^2^* FRT-2A/TM3, *Ser twist*>GFP males. Embryos resulting from germline clones were collected on egg lay plates, then analysed by confocal microscopy after mounting in DAPI-containing Vectashield medium. In positive controls where *Med19* ^+^ replaced *Med19^2^*, all expected zygotic classes were obtained as viable, fertile adults. In the absence of heat shock, no eggs were laid.

### Antibody staining

Performed as described in [40]. Antibodies used were: rat anti-Bab2 (J-L Couderc, used at 1∶3000); mouse anti-GFP (VC) (Roche, 1∶200) or chicken anti-GFP (VC) (Invitrogen); rabbit anti-Spalt (R. Barrio, 1∶100); mouse anti-Col (M. Crozatier/A. Vincent, 1∶200); mouse anti-dSRF (M. Affolter, 1∶1000); rabbit anti β-Gal (Cappel 1∶2500), mouse anti-Wg (1∶200) and anti-Ubx (1∶50) from the Developmental Studies Hybridoma Bank, University of Iowa.

### Anti-Med19 and -Med1 sera

Guinea pigs were immunized (Eurogentec) with GST-Med19 or GST-Med1 proteins extracted from *E. coli* and enriched by affinity chromatography. Anti-Med19 sera from terminal bleeds was used for immunocytology without purification at a 1∶500 dilution after prior pre-absorption on wild type larvae.

### Phenotypic analyses

Adult phenotypes were analyzed by light microscopy (Zeiss Axiophot) of dissected samples mounted in Hoyer's medium or by scanning electron microscopy (Hitachi TM-1000 Tabletop model) of frozen adults

### Sequence alignments

These were generated with the T-Coffee Program, employing the methodology described by [21].

## Supporting Information

Figure S1 Venus fusion proteins VN-Ubx and VN-Dfd are functional in the *Drosophila* embryo. Cuticles of wild-type embryos, or of embryos ectopically expressing Hox proteins. First line: left, wild-type embryo showing anterior cuticle from the head to abdominal segment 1 (A1). The three thoracic belts of fine denticles and the first band of denser abdominal denticles are indicated (T1, T2, T3 and A1, respectively); middle and right, similar transformations of T1, T2 and T3 denticle belts to A1 are induced by the ubiquitous expression of Ubx or of chimeric VN-Ubx, respectively, from UAS enhancers under arm-Gal4 control (arm>). Second line: left, wild-type embryonic head with cephalo-pharyngeal cuticle. Middle and right: *arm*-Gal4 driver-directed expression of Dfd or VN-Dfd from a UAS enhancer (arm>) results in similar, major defects of normal head structures, accompanied by the appearance of ectopic maxillary cirri (arrows and inset) typical of Dfd function.
(TIF)

Figure S2 Hexapeptide and linker region are dispensable for interaction with Med19. Bar drawings on the left represent (top) the HD region of *Drosophila* Ubx, with its hexapeptide (HX), linker region and HD; (middle) the HD region of *Drosophila* Ubx, but with its hexapeptide mutated (HXm) as described in Hudry et al (34); (right) the HD region of crustacean Artemia Ubx, whose HD is identical to the *Drosophila* sequence but whose linker region is much shorter. On the right, GST pulldowns show similar binding to wild-type Ubx (7% of input), Ubx whose HX is mutated (5%), or Artemia Ubx with shortened linker (5%).
(TIF)

Figure S3 Strong maternal effect of Med19 mutant germline clones. The photos in A, B and C present the cellular progression of embryos lacking maternally contributed Med19 (as seen by DAPI staining of nuclear DNA). (A,B) These embryos are pre-cellular, aged ≈1 hr and ≈2 hr, with the latter corresponding to the onset of zygotic transcription. (C) This embryo, seen shortly after cellularisation, shows massive disorganisation.
(TIF)

Figure S4 Med19 dsRNA affects the differentiation of larval posterior spiracles and mouthparts. Left column photos: wild-type larval posterior spiracles (top) and mouthparts (bottom). Right column photos: L3 larvae expressing UAS-dsRNA directed against Med19 under *daughterless*-Gal4 control (*da*>dsMed19^27559^). These photos reveal defects of posterior spiracles (above) or of larval mouthparts (bottom), resembling the embryonic defects of Hox mutants (noted to the right).
(TIF)

Figure S5 *Med19^2^* null clones can be rescued by UAS-Med19 transgene expression or in a Minute context. Mitotic clones homozygote for *Med19^2^* were induced in wing imaginal discs by *en*-Gal4 coupled with UAS-Flp (*en*>Flp) as described in text. (A, A′, A″): *en*-Gal4 also directed UAS-Med19-VC expression (*en*>Flp>Med19VC). A large −/− clone is detected by the absence of green GFP (A); Med19 and Med19-VC proteins are both detected by anti-Med19 sera (red, A′); the merged image is shown in A″. (B, B′, B″): Mitotic clones were induced as for A–A″. Rather than supply transgenic Med19, clones were induced in the presence of a *Minute* mutation on the homologous chromosome. (B) −/− clones are detected on the right-hand side of this wing imaginal disc by the absence of green GFP marker. (B′) Anti-Med19 sera (red) showed no signal in mutant cells. (B″) Merged images confirm the absence of red signal in mutant cells.
(TIF)

Figure S6 Table 1, interaction data. Phenotypic analyses indicate interactions of Med19 lof mutations with the *Antp* gof allele *Antp^Ns^*; with the *Antp* lof allele *Antp^Ns^* ^+RC3^; with the *Dfd* gof allele *Dfd^1^*; and with a lof combination for *Dfd* (*ptc*-Gal4>UAS-RNAi (Dfd)). The heterozygous presence of *Med19^2^* significantly altered the phenotypic outcome in each case.
(TIF)

Figure S7 The Med19 Hox “Homeodomain Interacting Motif” (HIM) is conserved across the animal kingdom. At top, a block representation of *Drosophila* Med19 indicates the internal location of the HIM element. Sequence alignments are shown for the species listed at the bottom.
(TIF)

Figure S8 Med19 variant incorporation into MED, expression levels and nuclear location. (A) Co-immunoprecipitation experiment. Extracts of *Drosophila* S2 cells transfected with act5C-Gal4 driver alone (control), with UAS-Med19-VC or - ΔHIM-VC plasmid, were immunoprecipitated with anti-GFP directed against the VC tag. Western blots of these precipitates tested with anti-Med1 revealed association of the three known Med1 isoforms (Input) with both Med19-VC and ΔHIM-VC in the presence of anti-GFP (IP GFP) but not in controls (IP). (B) Characterisation of expression levels and cellular localisation for Med19-VC, ΔHIM-VC and HIM-VC. (C,D,E) The three proteins are accumulated at similar levels when expressed under *dpp*-Gal4 control in wing imaginal discs, as seen with anti-GFP. (C) Med19-VC is expressed as a band in the wing imaginal disc under *dpp*-Gal4 control, detected here with anti-GFP. (F) Enlargement of the boxed region of C. reveals nuclear Med19-VC (arrow), that coincides with anti-Med19 staining (F′) and DAPI staining of nuclear DNA (F″). F″′ presents the merged signals. (D) ΔHIM-VC expressed in the wing imaginal disc under *dpp*-Gal4 control is detected with anti-GFP. (G–G″′) G, enlargement of the boxed region of D. A single representative cell (arrow) shows co-localisation for anti-GFP (G) and anti-Med19 (G′). As shown by DAPI (G″) and in the merged image (G″′), ΔHIM-VC is present both in the nucleus and the cytoplasm. (E) Expression of HIM-VC under *dpp*-Gal4 control, visualised in a wing imaginal disc with anti-GFP. (H) Enlargement of the boxed region of E. Nuclear HIM localisation (arrow) is confirmed in H′ (anti-Med19), H″ (DAPI) and in the merged image (H″′).
(TIF)

Figure S9 Table 2, phenotypic rescue by Med19-VC and ΔHIM-VC constructs. These forms of Med19 were employed to rescue effects of Med19 mutant combinations. Top: *arm*-Gal4 driver directed expression of UAS-Med19-VC and -ΔHIM-VC, in the pupal-lethal *Med19^1^*/*Med19^2^* context. Culture temperatures are noted. Adult viability was partially restored by Med19-VC, but not by ΔHIM-VC. Spiracle eversion and maxillary formation (where Mx* indicates a mal-formed adult palp) were rescued to a greater extent by Med19-VC. Bottom: −/− haltere clones were induced in the presence of a *Minute* mutation by *apterous*-Flp (*ap*-Flp), alone or in the presence of UAS-Med19-VC or -ΔHIM-VC. Only Med19-VC yielded apparent rescue.
(TIF)
