# Supplementary figures and images for: Drosophila melanogaster Hox Transcription Factors Access the RNA Polymerase II Machinery through Direct Homeodomain Binding to a Conserved Motif of Mediator Subunit Med19
Source: PLoS Genet. 2014 May 1;10(5):e1004303. doi: 10.1371/journal.pgen.1004303 (PMC4006704; doi:10.1371/journal.pgen.1004303)

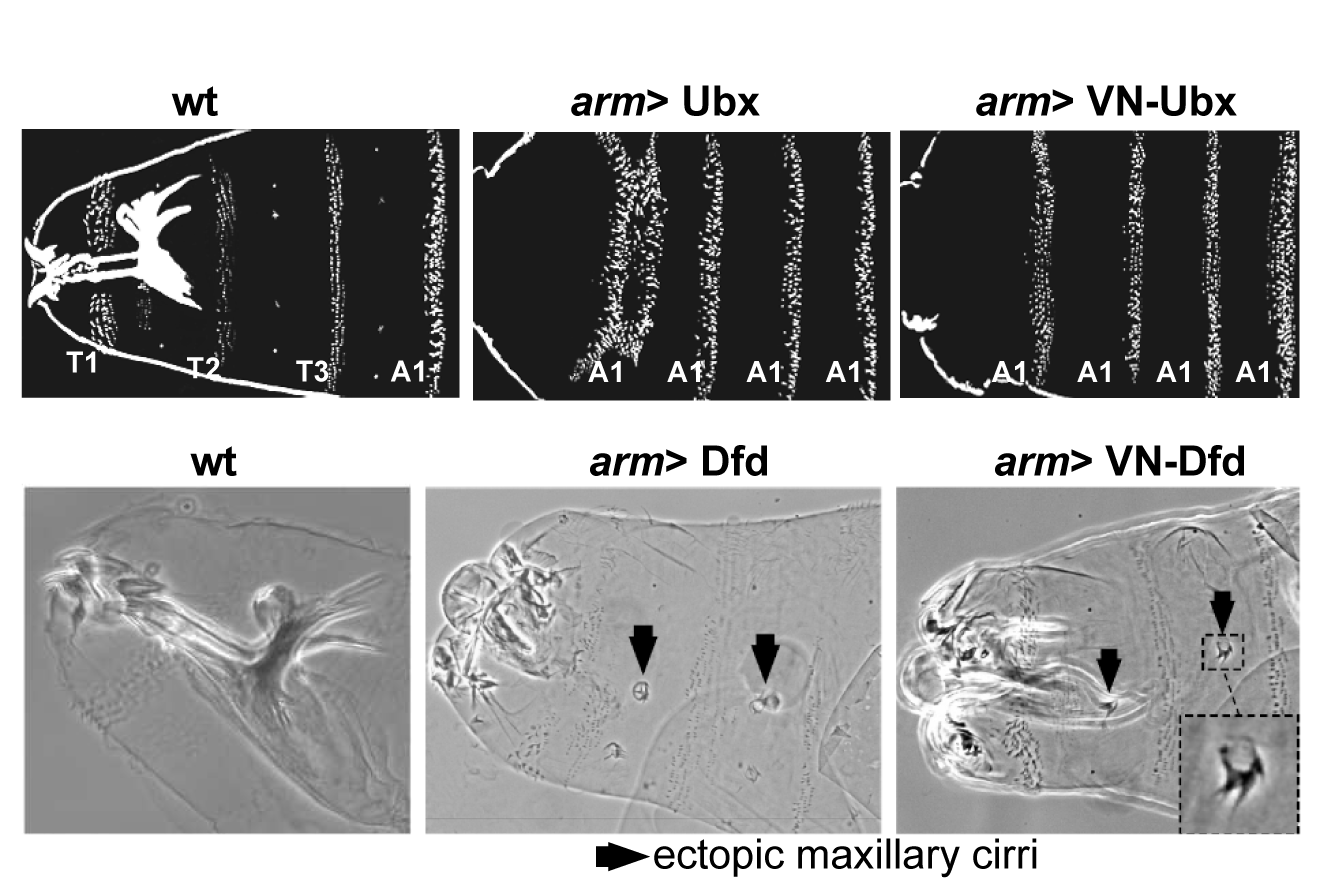

Supplement: Figure S1 — Venus fusion proteins VN-Ubx and VN-Dfd are functional in the Drosophila embryo. Cuticles of wild-type embryos, or of embryos ectopically expressing Hox proteins. First line: left, wild-type embryo showing anterior cuticle from the head to abdominal segment 1 (A1). The three thoracic belts of fine denticles and the first band of denser abdominal denticles are indicated (T1, T2, T3 and A1, respectively); middle and right, similar transformations of T1, T2 and T3 denticle belts to A1 are induced by the ubiquitous expression of Ubx or of chimeric VN-Ubx, respectively, from UAS enhancers under arm-Gal4 control (arm>). Second line: left, wild-type embryonic head with cephalo-pharyngeal cuticle. Middle and right: arm-Gal4 driver-directed expression of Dfd or VN-Dfd from a UAS enhancer (arm>) results in similar, major defects of normal head structures, accompanied by the appearance of ectopic maxillary cirri (arrows and inset) typical of Dfd function. (TIF) [file pgen.1004303.s001.tif]

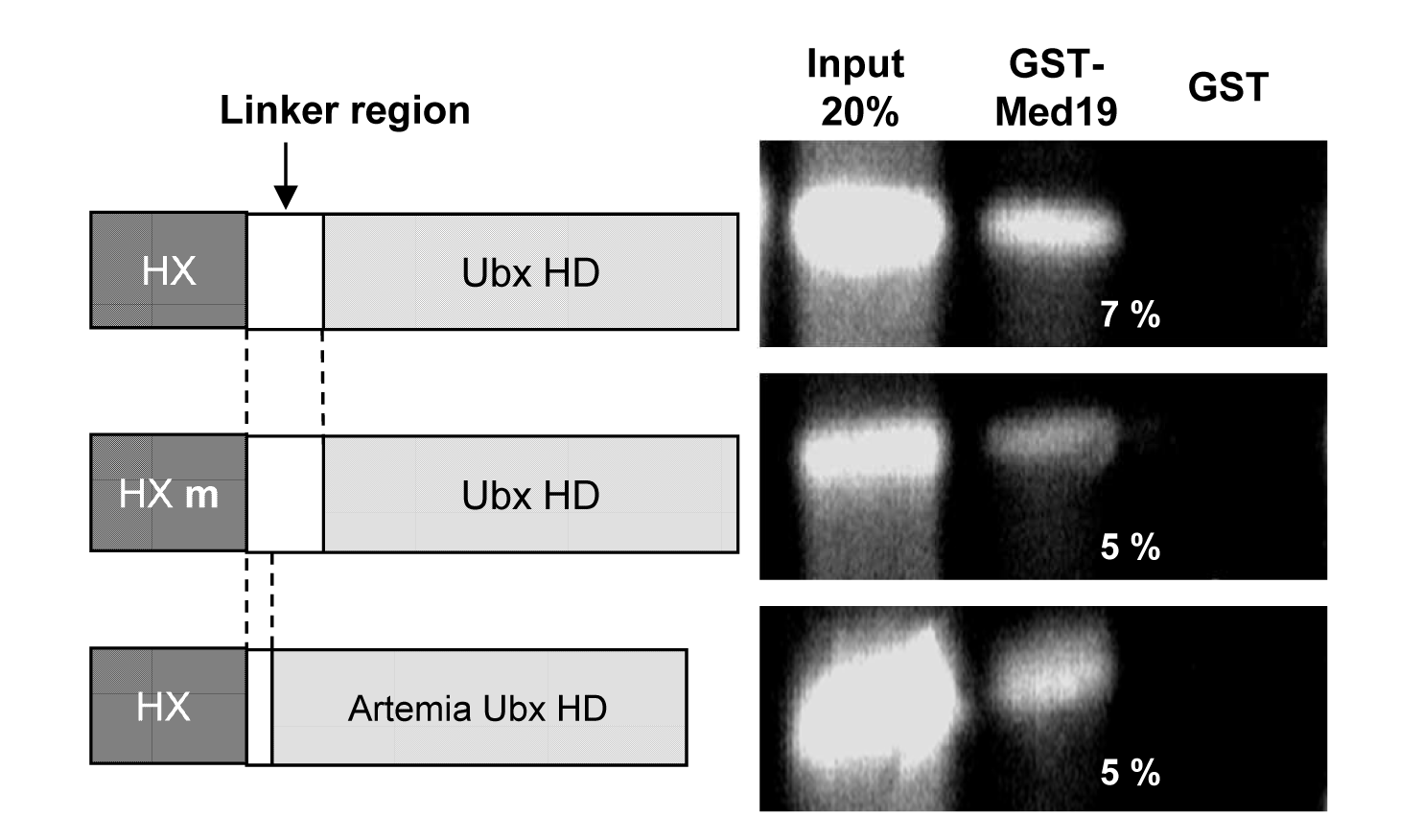

Supplement: Figure S2 — Hexapeptide and linker region are dispensable for interaction with Med19. Bar drawings on the left represent (top) the HD region of Drosophila Ubx, with its hexapeptide (HX), linker region and HD; (middle) the HD region of Drosophila Ubx, but with its hexapeptide mutated (HXm) as described in Hudry et al (34); (right) the HD region of crustacean Artemia Ubx, whose HD is identical to the Drosophila sequence but whose linker region is much shorter. On the right, GST pulldowns show similar binding to wild-type Ubx (7% of input), Ubx whose HX is mutated (5%), or Artemia Ubx with shortened linker (5%). (TIF) [file pgen.1004303.s002.tif]

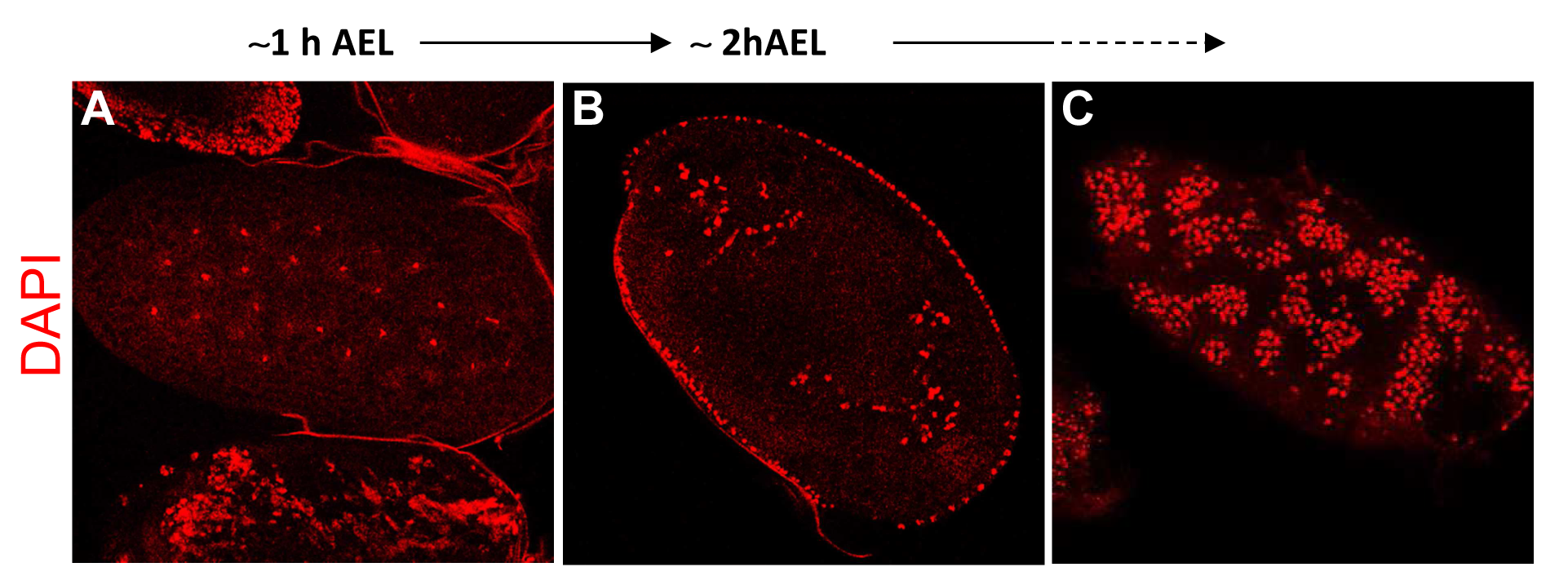

Supplement: Figure S3 — Strong maternal effect of Med19 mutant germline clones. The photos in A, B and C present the cellular progression of embryos lacking maternally contributed Med19 (as seen by DAPI staining of nuclear DNA). (A,B) These embryos are pre-cellular, aged ≈1 hr and ≈2 hr, with the latter corresponding to the onset of zygotic transcription. (C) This embryo, seen shortly after cellularisation, shows massive disorganisation. (TIF) [file pgen.1004303.s003.tif]

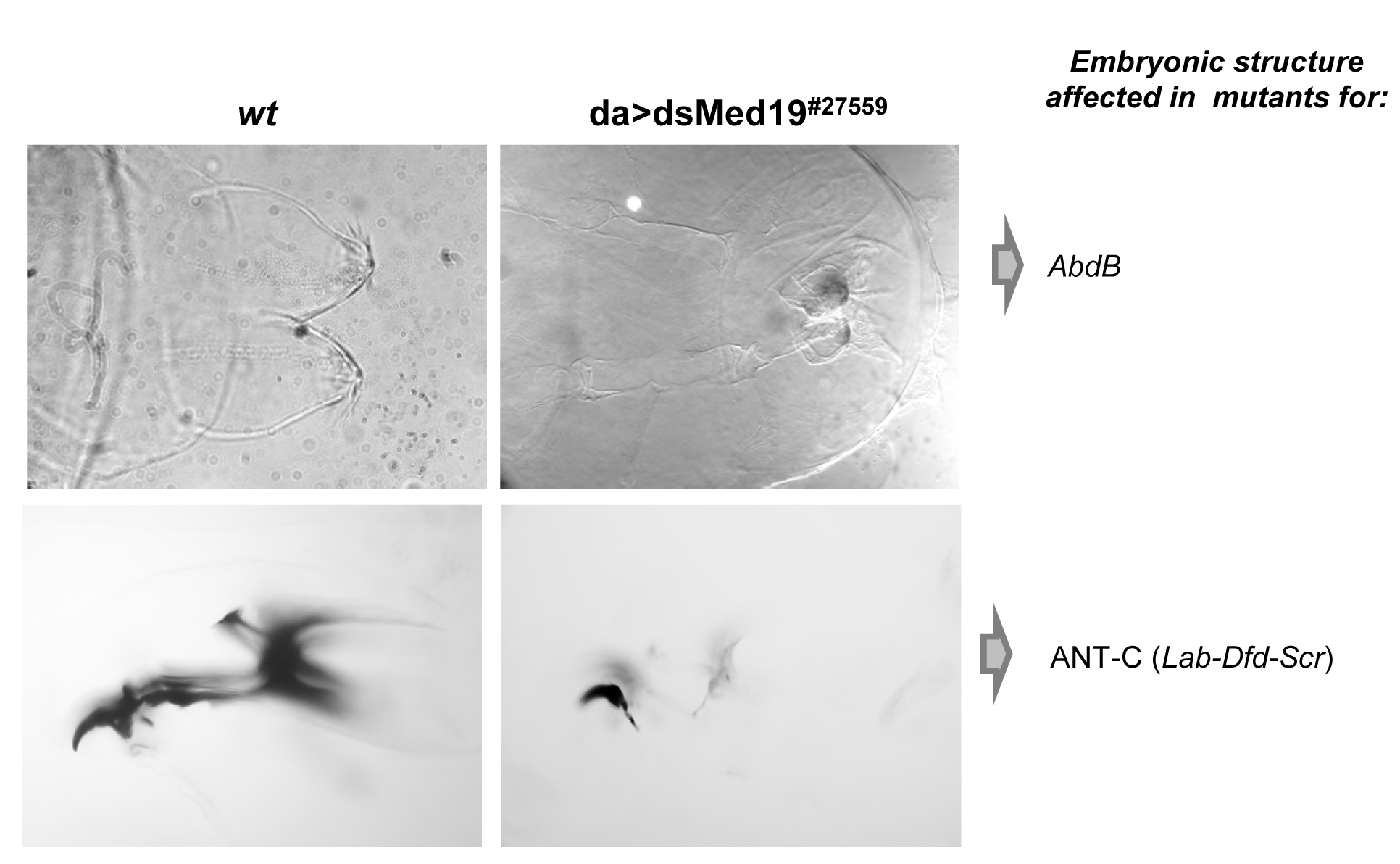

Supplement: Figure S4 — Med19 dsRNA affects the differentiation of larval posterior spiracles and mouthparts. Left column photos: wild-type larval posterior spiracles (top) and mouthparts (bottom). Right column photos: L3 larvae expressing UAS-dsRNA directed against Med19 under daughterless-Gal4 control (da>dsMed1927559). These photos reveal defects of posterior spiracles (above) or of larval mouthparts (bottom), resembling the embryonic defects of Hox mutants (noted to the right). (TIF) [file pgen.1004303.s004.tif]

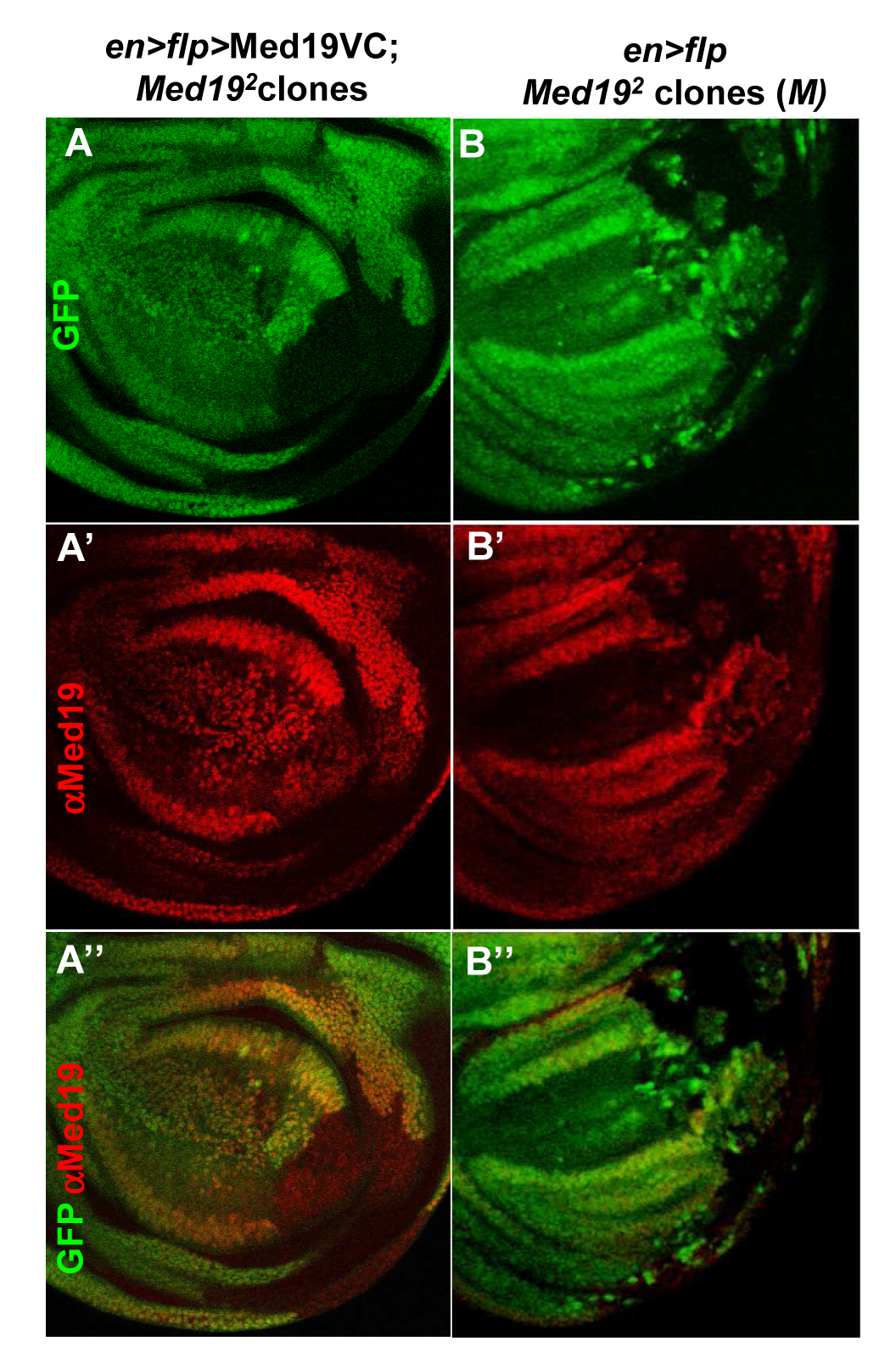

Supplement: Figure S5 — Med192 null clones can be rescued by UAS-Med19 transgene expression or in a Minute context. Mitotic clones homozygote for Med192 were induced in wing imaginal discs by en-Gal4 coupled with UAS-Flp (en>Flp) as described in text. (A, A′, A″): en-Gal4 also directed UAS-Med19-VC expression (en>Flp>Med19VC). A large −/− clone is detected by the absence of green GFP (A); Med19 and Med19-VC proteins are both detected by anti-Med19 sera (red, A′); the merged image is shown in A″. (B, B′, B″): Mitotic clones were induced as for A–A″. Rather than supply transgenic Med19, clones were induced in the presence of a Minute mutation on the homologous chromosome. (B) −/− clones are detected on the right-hand side of this wing imaginal disc by the absence of green GFP marker. (B′) Anti-Med19 sera (red) showed no signal in mutant cells. (B″) Merged images confirm the absence of red signal in mutant cells. (TIF) [file pgen.1004303.s005.tif]

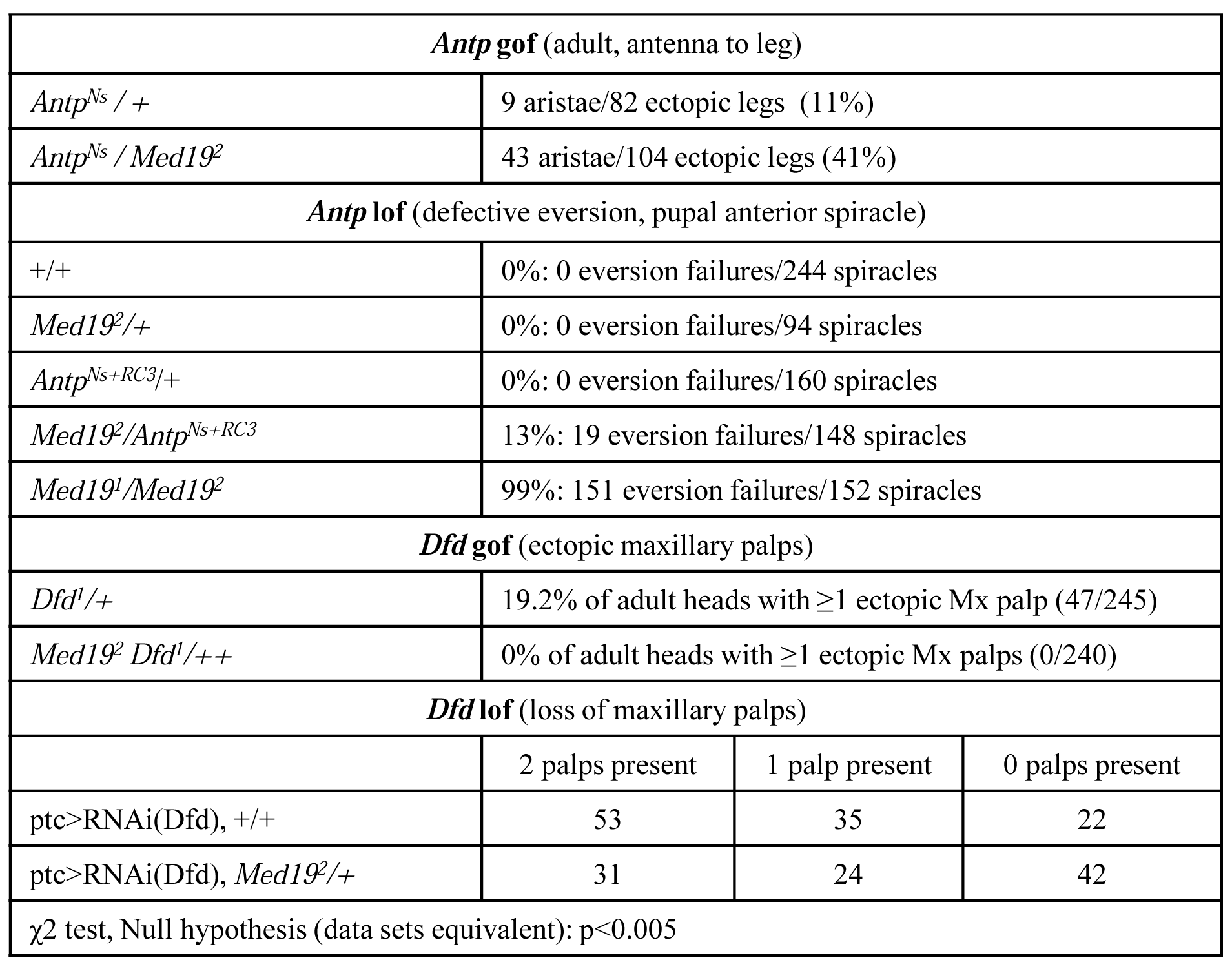

Supplement: Figure S6 — Table 1, interaction data. Phenotypic analyses indicate interactions of Med19 lof mutations with the Antp gof allele AntpNs; with the Antp lof allele AntpNs +RC3; with the Dfd gof allele Dfd1; and with a lof combination for Dfd (ptc-Gal4>UAS-RNAi (Dfd)). The heterozygous presence of Med192 significantly altered the phenotypic outcome in each case. (TIF) [file pgen.1004303.s006.tif]

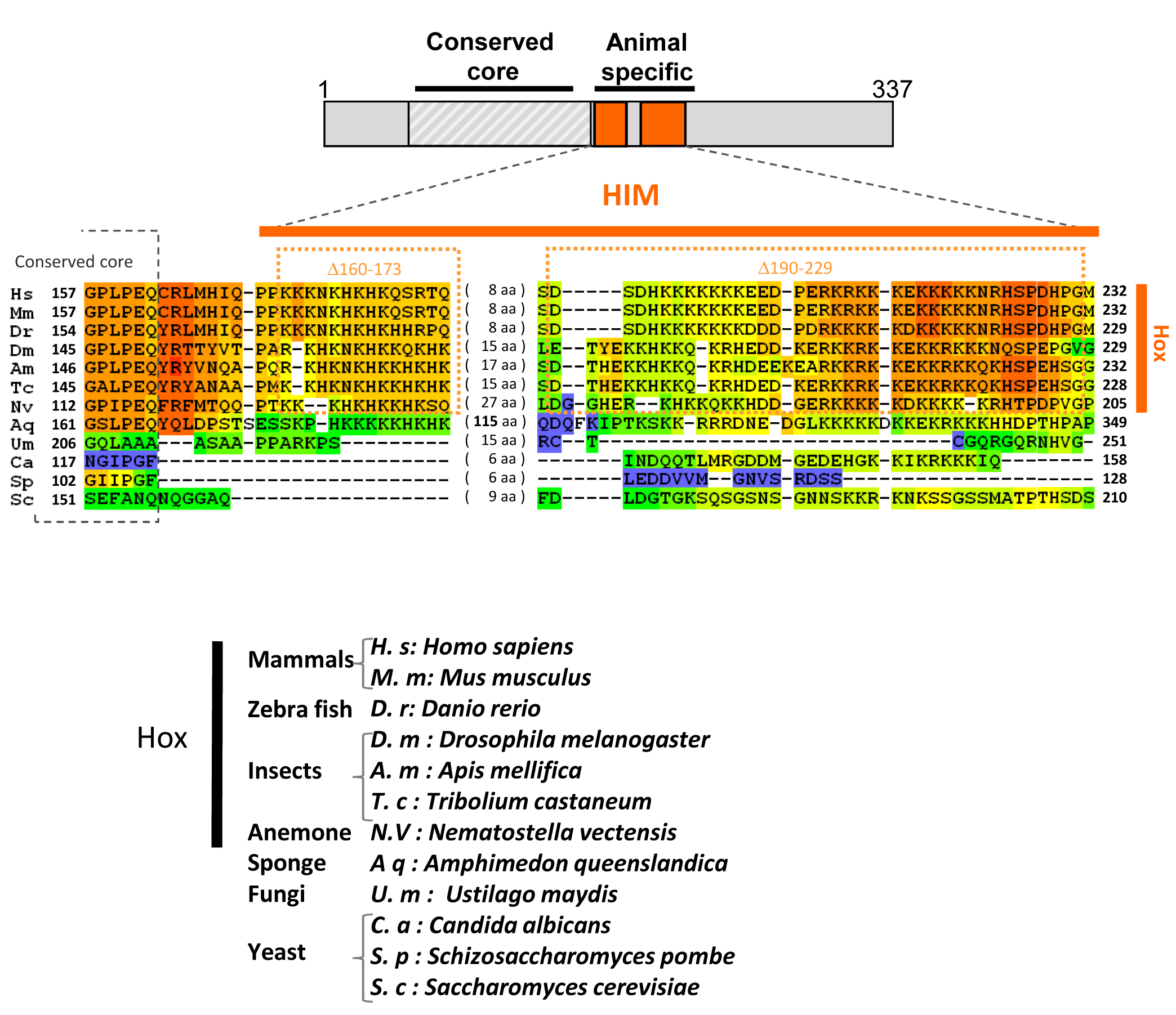

Supplement: Figure S7 — The Med19 Hox “Homeodomain Interacting Motif” (HIM) is conserved across the animal kingdom. At top, a block representation of Drosophila Med19 indicates the internal location of the HIM element. Sequence alignments are shown for the species listed at the bottom. (TIF) [file pgen.1004303.s007.tif]

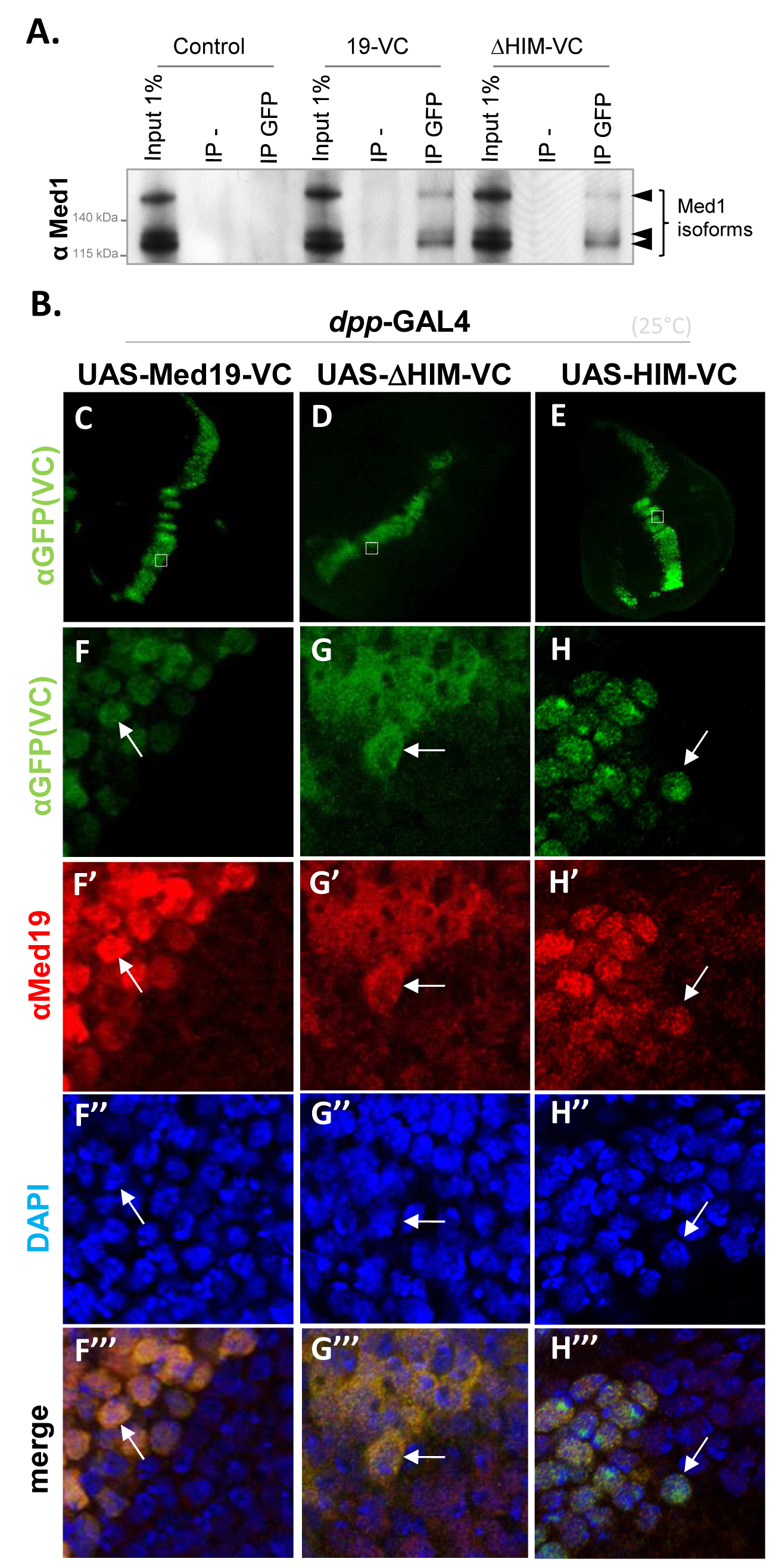

Supplement: Figure S8 — Med19 variant incorporation into MED, expression levels and nuclear location. (A) Co-immunoprecipitation experiment. Extracts of Drosophila S2 cells transfected with act5C-Gal4 driver alone (control), with UAS-Med19-VC or - ΔHIM-VC plasmid, were immunoprecipitated with anti-GFP directed against the VC tag. Western blots of these precipitates tested with anti-Med1 revealed association of the three known Med1 isoforms (Input) with both Med19-VC and ΔHIM-VC in the presence of anti-GFP (IP GFP) but not in controls (IP). (B) Characterisation of expression levels and cellular localisation for Med19-VC, ΔHIM-VC and HIM-VC. (C,D,E) The three proteins are accumulated at similar levels when expressed under dpp-Gal4 control in wing imaginal discs, as seen with anti-GFP. (C) Med19-VC is expressed as a band in the wing imaginal disc under dpp-Gal4 control, detected here with anti-GFP. (F) Enlargement of the boxed region of C. reveals nuclear Med19-VC (arrow), that coincides with anti-Med19 staining (F′) and DAPI staining of nuclear DNA (F″). F″′ presents the merged signals. (D) ΔHIM-VC expressed in the wing imaginal disc under dpp-Gal4 control is detected with anti-GFP. (G–G″′) G, enlargement of the boxed region of D. A single representative cell (arrow) shows co-localisation for anti-GFP (G) and anti-Med19 (G′). As shown by DAPI (G″) and in the merged image (G″′), ΔHIM-VC is present both in the nucleus and the cytoplasm. (E) Expression of HIM-VC under dpp-Gal4 control, visualised in a wing imaginal disc with anti-GFP. (H) Enlargement of the boxed region of E. Nuclear HIM localisation (arrow) is confirmed in H′ (anti-Med19), H″ (DAPI) and in the merged image (H″′). (TIF) [file pgen.1004303.s008.tif]

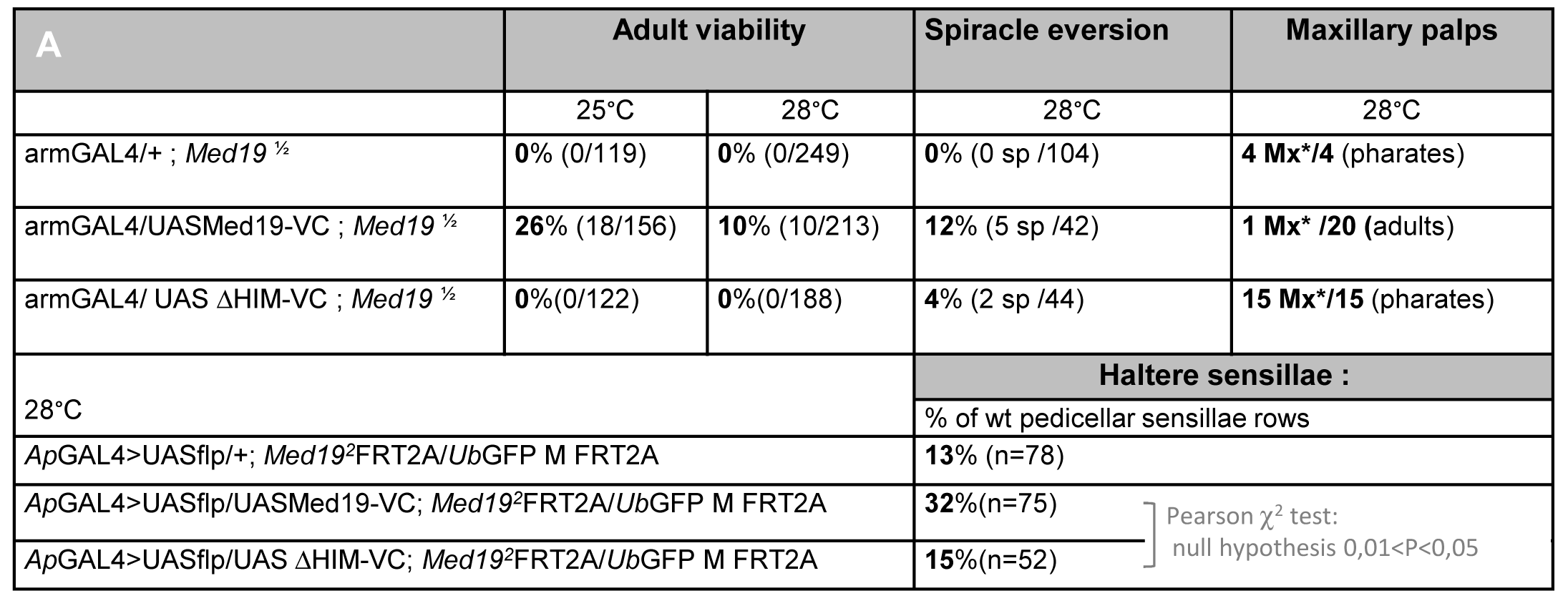

Supplement: Figure S9 — Table 2, phenotypic rescue by Med19-VC and ΔHIM-VC constructs. These forms of Med19 were employed to rescue effects of Med19 mutant combinations. Top: arm-Gal4 driver directed expression of UAS-Med19-VC and -ΔHIM-VC, in the pupal-lethal Med191/Med192 context. Culture temperatures are noted. Adult viability was partially restored by Med19-VC, but not by ΔHIM-VC. Spiracle eversion and maxillary formation (where Mx* indicates a mal-formed adult palp) were rescued to a greater extent by Med19-VC. Bottom: −/− haltere clones were induced in the presence of a Minute mutation by apterous-Flp (ap-Flp), alone or in the presence of UAS-Med19-VC or -ΔHIM-VC. Only Med19-VC yielded apparent rescue. (TIF) [file pgen.1004303.s009.tif]
